# The GATA3 X308_Splice breast cancer mutation is a hormone context-dependent oncogenic driver

**DOI:** 10.1038/s41388-020-1376-3

**Published:** 2020-06-25

**Authors:** Natascha Hruschka, Mark Kalisz, Maria Subijana, Osvaldo Graña-Castro, Francisco Del Cano-Ochoa, Laia Paré Brunet, Igor Chernukhin, Ana Sagrera, Aurelien De Reynies, Bernhard Kloesch, Suet-Feung Chin, Octavio Burgués, David Andreu, Begoña Bermejo, Juan Miguel Cejalvo, Joe Sutton, Carlos Caldas, Santiago Ramón-Maiques, Jason S. Carroll, Aleix Prat, Francisco X. Real, Paola Martinelli

**Affiliations:** 1 Institute of Cancer Research, Medical University Vienna, Comprehensive Cancer Center, Vienna, Austria; 2 Epithelial Carcinogenesis Group, Spanish National Cancer Research Centre-CNIO, CIBERONC, Madrid, Spain; 3 Bioinformatics Unit, Spanish National Cancer Research Centre-CNIO, Madrid, Spain; 4 Department of Genome Dynamics and Function, Centro de Biología Molecular Severo Ochoa (CSIC-UAM), Madrid, Spain; 5 Department of Medical Oncology, Hospital Clínic, Barcelona, Spain; 6 Translational Genomics and Targeted Therapeutics in Solid Tumors, IDIBAPS, Barcelona, Spain; 7 Cancer Research UK Cambridge Institute, University of Cambridge, Robinson Way, Cambridge, CB2 ORE UK; 8 Programme Cartes d’Identité des Tumeurs, Ligue Nationale Contre le Cancer, 75013 Paris, France; 9 Department of Oncology, Cancer Research UK Cambridge Institute, University of Cambridge, Cambridge, UK; 10 INCLIVA Biomedical Research Institute, Valencia, Spain; 11 Pathology Department, Hospital Clínico Universitario-CIBERONC, Valencia, Spain; 12 Laboratory of Proteomics and Protein Chemistry, Universitat Pompeu Fabra, Barcelona, Spain; 13 Oncology and Hematology Department, Hospital Clínico Universitario-CIBERONC, Valencia, Spain; 14 Departament de Ciències Experimentals i de la Salut, Universitat Pompeu Fabra, Barcelona, Spain; 15 Present Address: Cancer Cell Signaling Department, Boehringer-Ingelheim RCV, Vienna, Austria

**Keywords:** Breast cancer, Cancer genetics

## Abstract

As the catalog of oncogenic driver mutations is expanding, it becomes clear that alterations in a given gene might have different functions and should not be lumped into one class. The transcription factor *GATA3* is a paradigm of this. We investigated the functions of the most common *GATA3* mutation (X308_Splice) and five additional mutations, which converge into a neoprotein that we called “neoGATA3,” associated with excellent prognosis in patients. Analysis of available molecular data from >3000 breast cancer patients revealed a dysregulation of the ER-dependent transcriptional response in tumors carrying neoGATA3-generating mutations. Mechanistic studies in vitro showed that neoGATA3 interferes with the transcriptional programs controlled by estrogen and progesterone receptors, without fully abrogating them. ChIP-Seq analysis indicated that ER binding is reduced in neoGATA3-expressing cells, especially at distal regions, suggesting that neoGATA3 interferes with the fine tuning of ER-dependent gene expression. This has opposite outputs in distinct hormonal context, having pro- or anti-proliferative effects, depending on the estrogen/progesterone ratio. Our data call for functional analyses of putative cancer drivers to guide clinical application.

## Introduction

Genomics studies have produced an expanding catalog of cancer-driving somatic mutations, which needs to be translated into biological and clinically applicable knowledge [1]. One limitation of many studies is the tendency to lump all mutations occurring in one gene into a single class, which can lead to inconclusive results when stratifying patients in a binary fashion, as different genetic alterations might have distinct effects [2–4]. The *GATA3* transcription factor is emerging as a paradigm of a gene where multiple classes of mutations occur, having distinct biological and clinical output [5–8]. This is specific for breast cancer (BC), where *GATA3* is mutated in around 11% of cases and shows a characteristic mutational pattern, different from other tumor types [2, 3].

**Fig. 1:**
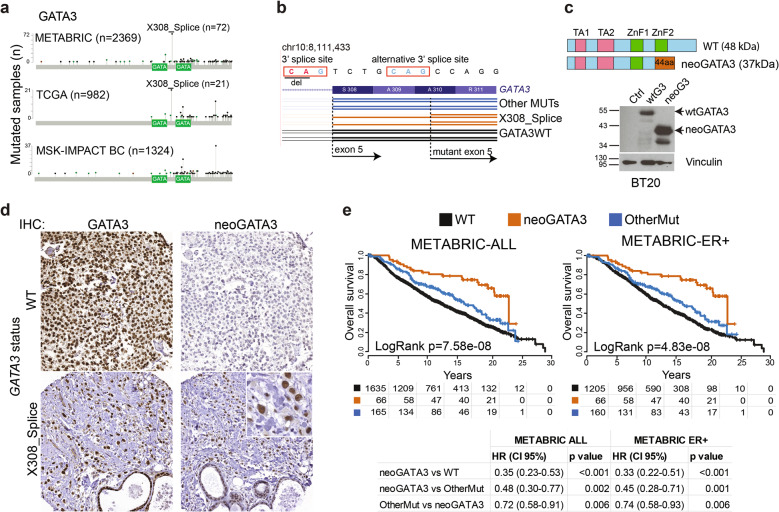
A hotspot splice-disrupting *GATA3* mutation correlates with good outcome in breast cancer. **a** Distribution of the *GATA3* mutations in the METABRIC, TCGA-BRCA, and MSK-IMPACT cohorts (only BC patients are shown for the latter). The two GATA boxes indicate the two Zn finger DNA-binding domains of the GATA3 protein. **b** Scheme of the mutant transcript identified in tumors carrying the X308_Splice mutation, compared with tumors with wt *GATA3* or with any other *GATA3* mutation. **c** Top: schematic representation of wt GATA3, compared with the predicted neoGATA3 protein. Bottom: western blot showing the expression of wild type GATA3 (wtG3) and the mutant neoGATA3. Black arrows indicate the proteins of the expected size. **d** Representative IHC images using either the N-ter GATA3 antibody—recognizing both wt and mutant GATA3 (left)—or the neoGATA3-specific antibody (right) on tumors carrying wild type GATA3 (top), or the X308_Splice mutation (bottom). **e** Kaplan–Meier survival curves of the METABRIC patients stratified according to *GATA3* status (WT = wild type, neoGATA3 = all mutations producing a neoGATA3-like peptide, OtherMut = all other mutations in *GATA3*). Left: all patients, right: ER + patients.

Several evidences implicate GATA3 in the activation of the mammary differentiation program: (1) in normal tissue, it is necessary for the luminal compartment formation [9]; (2) GATA3 expression in BC strongly correlates with estrogen receptor (ER) expression [10]; (3) GATA3 functions in a complex with FOXA1 and ER to enhance transcription of ER-responsive genes [11]; and (4) ectopic expression in GATA3-negative basal-like BC cells is sufficient to induce luminal differentiation and inhibit tumor dissemination [12]. Consistently, GATA3 expression decreases during progression to metastatic BC [13]. The high frequency of *GATA3* mutations in BC suggests that they are driver mutations, but whether they result in loss-of-function (LOF) or gain-of-function (GOF) is not clear. Most *GATA3* mutations are rare or unique frameshift indels (insertion/deletions) distributed along the 3′ gene end (Fig. 1a), consistent with the classical mutational pattern of a tumor suppressor and therefore suggesting a LOF [2]. However, they are typically heterozygous and the expression of the wild type (WT) allele is retained [14]. A few mutations concentrate in two clusters in exon 5 and 6, including some “hotspots” or “warmspots,” suggesting that they might generate GOF, instead. Whether *GATA3* mutations are true oncogenic drivers is also an open question: while some in vitro and in vivo data suggest a tumor-promoting function [6, 8, 15], in general they are associated with longer survival [2] and better response to endocrine therapy [16]. A recent study identified four classes of *GATA3* frameshift mutations: (1) ZnFn2 mutations, occurring within the C-terminal Zn finger; (2) splice mutations, occurring mainly between intron 4 and exon 5; (3) truncating mutations, occurring downstream of the C-terminal Zn finger; and (4) extension mutations, occurring in exon 6 and disrupting the stop codon [6]. ZnFn2 mutations produce a highly stable truncated protein lacking the C-terminal Zn finger, showing low affinity for DNA and altered transcriptional activity, and are associated with poor outcome when compared with other *GATA3* mutations [6, 17]. Extension mutations produce a longer protein modulating drug sensitivity [5]. The effect of splice and truncating mutations remains unknown.

Here, we investigated the effects of the most prevalent *GATA3* hotspot somatic mutation (X308_Splice). This mutation, like five additional ones producing partially or fully identical C-terminal peptides, correlates with better outcome in patients and is associated with a specific gene expression signature, characterized by altered ER-dependent transcriptional program. Combined analysis of patient-derived data and in vitro experiments with BC cell lines shows that the mutant protein—which we designate as “neoGATA3”—interferes with the function of both ER and PR, blunting, without abrogating, their downstream programs. This has distinct biological outputs depending on the hormonal context: neoGATA3-expressing cells have a proliferative advantage when both estrogen and progesterone levels are high while they display a growth disadvantage when estrogen prevails. Our data suggest the existence of stage-dependent oncogenic effects of GATA3 driver mutations.

## Results

### The *GATA3* X308_splice mutation produces a unique neopeptide

The most common *GATA3* mutation is a 2nt deletion in intron 4 disrupting the 3′ splice site (X308_Splice, Fig. 1a). The predicted effect is a transcript lacking 7 nucleotides [7, 14] which we successfully identified in RNA-Seq data from 15/19 TCGA-BRCA samples carrying the X308_Splice mutation but not in 20/20 tumors with either WT *GATA3* or other *GATA3* mutations (Fisher’s exact probability test *P* = 1.54e−07, Fig. 1b). The mutant transcript was validated by RT-qPCR in 4/4 independent luminal A/B tumors carrying the X308_Splice mutation and in 0/7 without it (Supplementary Fig. 1a). The loss of 7nt causes a frameshift, leading to a GATA3 protein—designated neoGATA3—lacking residues 308–444, encompassing the second ZnFn, and containing instead a novel 44aa C-terminal sequence without homology to any other human protein sequence (Fig. 1c). We raised a polyclonal antiserum against the novel 44aa peptide, which specifically recognized a shorter GATA3 protein of the expected size (37 kDa) exclusively in a tumor carrying the mutation and in cells transduced with the mutant cDNA (Fig. 1c and Supplementary Fig. 1b). These antibodies allowed the detection of neoGATA3 in a tissue microarray containing 100 luminal A/B tumors with information about *GATA3* mutational status with high sensitivity (90%) and specificity (94%) (Fig. 1d).

Importantly, we identified five additional mutations, detected in six METABRIC and one TCGA-BRCA samples, producing fully or partially identical C-terminal peptides. One of them is a 2nt insertion at codon Q321, found after re-sequencing one METABRIC sample originally genotyped as *GATA3*-WT (MB-0114) showing immunoreactivity with the mutant-specific antibodies (Supplementary Fig. 1c). In all, at least six different mutations, found in 78/2369 (3.3%) METABRIC samples and in 22/988 (2.2%) TCGA-BRCA samples, produce a neoGATA3-like peptide (Supplementary Table 1).

### Patients with neoGATA3-mutant tumors display excellent prognosis

To understand the clinical significance of neoGATA3 mutations, we analyzed the METABRIC cohort, where clinical data are available for 1673 patients, including 231 (13.8%) with *GATA3*-mutant tumors. Among the latter, 66 (28.6%) had neoGATA3-type mutations and 165 (71.4%) had other mutations. NeoGATA3 mutations were significantly associated with lower tumor stage, grade, and size and with expression of progesterone receptor (PR) (Supplementary Fig. 2a–d), all factors predicting better outcome. Consistently, patients with neoGATA3-mutant tumors had significantly better overall survival (OS) compared to both patients with WT *GATA3* (HR = 0.35; 95% CI 0.23–0.53; *P* < 0.001) and those carrying any other *GATA3* mutation (OtherMut) (HR = 0.48; 95% CI 0.30–0.77; *P* = 0.002, Fig. 1e, left).

Importantly, neoGATA3 mutations were exclusive for patients with ER + tumors (Supplementary Fig. 3a), which have better outcome [3, 18]. Even within this good-prognosis group, the presence of neoGATA3 mutations was strongly associated with significantly longer OS (neoGATA3 vs WT HR = 0.33; 95% CI 0.22–0.51; *P* < 0.001 and neoGATA3 vs OtherMut HR = 0.45; 95% CI 0.28–0.72; *P* = 0.001, Fig. 1e, right). A tendency toward longer disease-free survival (DFS) was observed for patients with neoGATA3-mutant tumors in the TCGA-BRCA ER + cohort although the differences were not statistically significant, likely due to smaller sample size (Supplementary Fig. 3b). Univariate and multivariate analyses showed that neoGATA3 is an independent prognostic factor of longer OS and disease-specific survival (DSS) in the METABRIC cohort (OS: HR = 0.58; 95% CI 0.36–0.92; *P* = 0.02; DSS: HR = 0.46; 95% CI 0.23–0.94; *P* = 0.03; Supplementary Tables 2 and 3). Consistently, none of the 1324 patients with metastatic BC included in the MSK-IMPACT cohort harbored neoGATA3 mutations [19] indicating that tumors with these mutations only metastasize exceptionally (*P* < 0.0001, Fig. 1a).

Strikingly, approximately one-third of the neoGATA3 mutations occurred in METABRIC ER + patients below 50 years (Supplementary Fig. 3c, *P* = 0.0004), suggesting that the effect of the neoGATA3 mutations might be affected by age or age-related factors, including the hormonal context.

To get insight into the molecular features of tumors harboring neoGATA3 mutations, we derived a gene expression signature based on a training set of 981 TCGA-BRCA samples (19 neoGATA3 mutations). This signature could identify neoGATA3-mutant tumors from the METABRIC series (*n* = 2001 samples with expression and mutation data, 63 neoGATA3) with a sensitivity of 68.3% and specificity of 80.5% both when applied as a continuous variable and as binary classifier. When the signature was used to classify the samples from a cohort of patients with no available mutational data [20], patients with tumors classified as positive for the neoGATA3-signature (either as continuous or binary classifier) showed significantly longer DFS (LogRank *P* = 0.004, not shown).

### Tumors with neoGATA3 mutations show changes in the immune microenvironment, not consistent with a T-cell mediated immune response

**Fig. 2:**
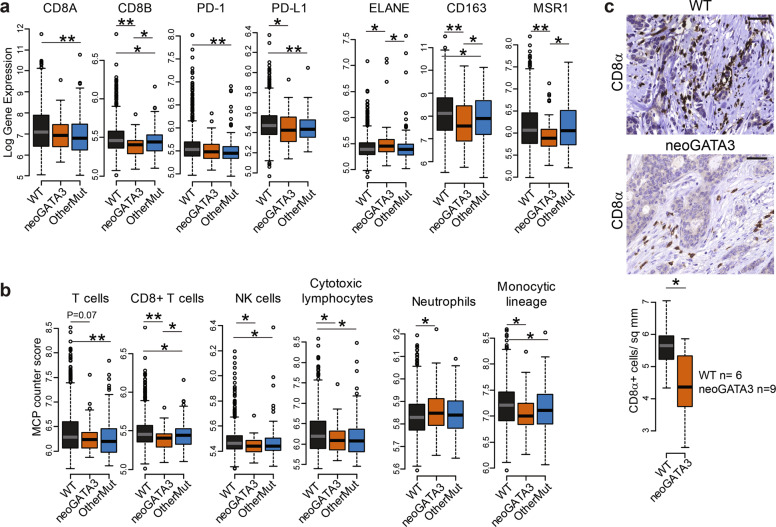
NeoGATA3-mutant tumors do not display a prominent immune response. **a** Gene expression levels in tumors of the METABRIC ER + cohort, divided in the three groups according to *GATA3* status (WT *n* = 1189, neoGATA3 *n* = 66, OtherMut *n* = 155). **b** MCP counter scores for the indicated immune cell populations in the three groups of METABRIC ER + tumors (WT *n* = 1205, neoGATA3 *n* = 65, OtherMut *n* = 161). **c** Representative IHC images of CD8α-positive cells in one tumor with wt GATA3 and in one with neoGATA3. Quantification of the staining of WT (*n* = 6) and neoGATA3 (*n* = 9) tumors is shown under the microphotographs. Mann–Whitney *U* test was used in (**a**, **b**), two-sided Student’s *t* test was used in (**c**) *P < 0.05, **P < 0.01.

A recent study identified the neopeptide of neoGATA3 as a potential neoantigen and suggested that it might induce an antitumor T-cell-dependent immune response and the activation of immune checkpoints [21]. To verify this hypothesis, we checked the expression of a set of markers of T-cell response in the METABRIC samples. Since neoGATA3 mutations are exclusive for ER + tumors, we only included these tumors in all our analyses. The expression of the T-cell marker CD8B (Mann–Whitney *U* test *P* = 0.001), and of the immune checkpoint protein PD-L1 (*P* = 0.033) was lower in neoGATA3 compared with WT tumors, while no significant difference was observed for CD8A and PD1 (Fig. 2a). To have a broader view of the immune landscape of neoGATA3 tumors, we used MCP counter to deconvolute the expression of immune markers and estimate the abundance of different cell populations [22]. A significant decrease in “CD8 + T cell” (*P* = 0.0003), “NK cell” (*P* = 0.01), and “Cytotoxic lymphocyte” (*P* = 0.021) signatures was observed in the neoGATA3 tumors when compared with the WT tumors while the “T cell” signature was unchanged (Fig. 2b). We then analyzed the amount of CD8 + T cells in a set of FFPE sections from WT (*n* = 6) and neoGATA3 (*n* = 9) tumors by IHC for CD8α protein. In accordance to gene expression data, CD8 + cells were significantly less abundant in neoGATA3 tumors (Fig. 2c, *P* = 0.048). No significant differences at the single gene level were observed in the neoGATA3 tumors of the TCGA cohort (Supplementary Fig. 4a) but the “C4-lymphocyte depleted” immunoscore [23] was overrepresented among the neoGATA3 tumors (4/17 neoGATA3 versus 47/525 WT), although statistical significance was not reached (*P* = 0.06, not shown).

In addition, the neutrophil marker ELANE (*P* = 0.011) was significantly increased, whereas the M2-macrophage markers CD163 (*P* = 0.0002) and MSR1 (coding for CD204, *P* = 0.003) were decreased in the neoGATA3 METABRIC tumors compared with WT (Fig. 2a) and showed similar tendencies in the TCGA-BRCA samples (Supplementary Fig. 4b). For some of the indicated markers, a significant difference was also observed when comparing neoGATA3 with OtherMut tumors (Fig. 2a, b). Although the differences between neoGATA3 and OtherMut tumors were rather small compared to the WT tumors, these observations suggest that the different mutations associate with distinct immune signatures. Consistent with these data, the MCP counter analyses revealed that the “Neutrophil” signature was upregulated (*P* = 0.04) and the “Monocytic lineage” signature was downregulated (*P* = 0.001) in the neoGATA3 tumors compared with WT, suggesting a complex modulation of the immune landscape in tumors carrying neoGATA3 mutations (Fig. 2b).

### Tumors with neoGATA3 mutations show decreased cell cycle progression and altered ER- and PR-dependent programs

**Fig. 3:**
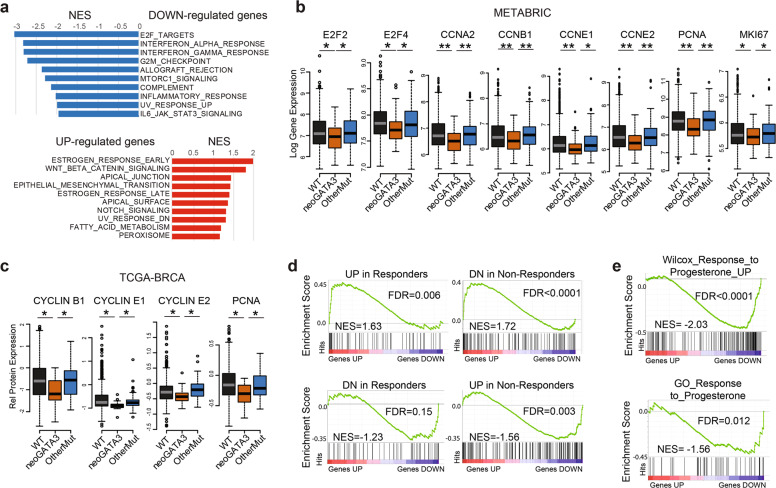
NeoGATA3 is associated with altered ER-dependent transcriptional program. **a** GSEA on the ranked list of genes differentially regulated in neoGATA3 tumors (*n* = 65) compared with all other tumors of the METABRIC ER + cohort (*n* = 1345). The “Hallmarks” collection of gene sets was interrogated. The graphs show the normalized enrichment score (NES) of the ten gene sets most significantly enriched among the upregulated (red) and the downregulated (blue) genes. FDR < 0.05 for all gene sets shown. **b** Gene expression data for the indicated cell cycle-related genes in the METABRIC ER + patients belonging to the three groups (WT *n* = 1189, neoGATA3 *n* = 66, OtherMut *n* = 155). **c** RPPA data from the TCGA cohort showing expression levels of the indicated proteins in the three tumor groups (WT *n* = 533, neoGATA3 *n* = 18, OtherMut *n* = 75). **d** Enrichment plots for genesets defined by Ross-Innes et al. comparing gene expression in tumors responding to endocrine therapy and tumors with poor response. GSEA was performed on genes differentially regulated in the neoGATA3 tumors from the METABRIC ER + cohort. **e** Enrichment plots for two progesterone-related genesets among the differentially expressed genes in the METABRIC neoGATA3 patients compared to all other METABRIC ER+. Mann–Whitney *U* test was used in (**b**, **c**). *P < 0.05, **P < 0.01.

Gene set enrichment analysis (GSEA) of the genes differentially expressed in the neoGATA3 tumors compared with all other tumors, revealed a strong downregulation of cell cycle- and inflammation-related Hallmarks gene sets (Fig. 3a). Accordingly, mRNA levels of E2F2 and E2F4, several cyclins, PCNA, and MKI67 were lower in the neoGATA3 METABRIC tumors (Fig. 3b) consistent with the better prognosis observed in patients. Similar differences were observed at the protein level in the TCGA series with reverse-phase protein arrays (RPPA) (Fig. 3c). This was not observed in tumors with other GATA3 mutations (Fig. 3b, c).

Among the genes upregulated in neoGATA3 tumors, we identified a significant enrichment of gene sets relative to estrogen response (both early and late), WNT/β-catenin signaling, and apical junctions (Fig. 3a). Interestingly, the neoGATA3-associated transcriptome showed positive correlation with a published gene signature of good prognosis after endocrine therapy and negative correlation with a signature of bad prognosis in BC patients [24] (Fig. 3d).

Recent work showed that the ZnFn2 *GATA3* mutations interfere with the expression of the *PGR* gene, coding for PR [6], while we observed that neoGATA3 mutations were associated with PR expression in tumors. We then examined several progesterone-related gene signatures and observed a downregulation in neoGATA3 tumors (Supplementary Fig. 5a) even when restricting the analysis to premenopausal patients, having higher progestogens levels (Fig. 3e). Interestingly, PGR was higher in neoGATA3 premenopausal tumors, compared to both WT (*P* = 0.01) and OtherMut (*P* = 0.005) (Supplementary Fig. 5b).

### The neoGATA3 protein is more stable and shows altered DNA binding

To investigate the molecular mechanisms underlying the association of neoGATA3 mutations with good prognosis, we searched for cellular models carrying the X308_Splice mutation. None of the analyzed 36 BC cell lines, which included 10 ER + lines, harbored this mutation, suggesting that cells with neoGATA3 mutations do not grow well in vitro. A collection of 90 PDX established from 81 patients was available [25]. While four of them carried a *GATA3* mutation, it was never neoGATA3, indicating that tumors carrying these mutations are not efficiently established in mice. We then attempted to introduce the mutation in the endogenous locus of luminal BC cell lines, but failed to identify any successfully engineered clone among the 50 analyzed. Another report mentioned the difficulty of generating knock-in clones with a different *GATA3* mutation [5] supporting a general lower fitness of *GATA3*-mutant BC cells in vitro. We therefore relied on lentiviral-based transduction of BC cells with HA-tagged neoGATA3 cDNA (HA-neoG3) and used Flag-tagged WT GATA3 cDNA (Flag-wtG3) or an empty vector as controls, to account for the effect of increased GATA3 protein dosage following neoGATA3 overexpression.

**Fig. 4:**
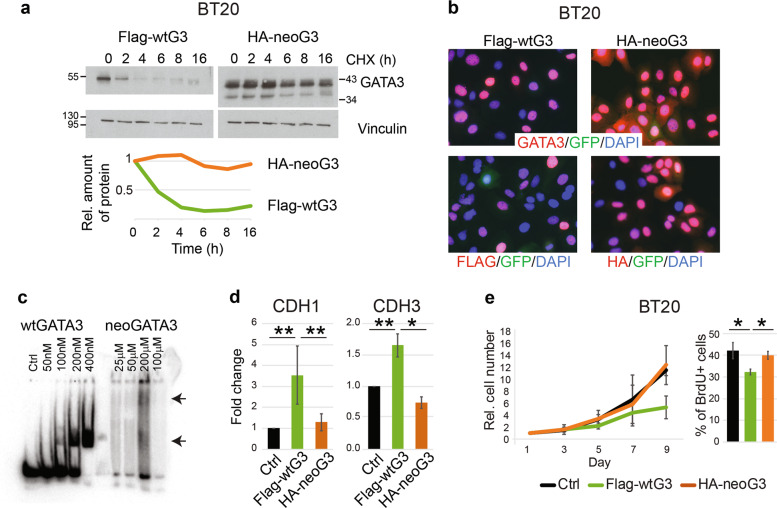
Biochemical and functional characteristics of neoGATA3 differ from wtGATA3. **a** Western blot showing the expression of wtGATA3 or neoGATA3 upon gene transduction in the GATA3-negative BT20 cells, after treatment with 50 µg/ml cycloheximide (CHX) for the indicated time. Vinculin was used as loading control. Quantification of relative band intensity is shown at the bottom. Images are representative of at least three independent experiments. **b** Immunofluorescence using the N-ter GATA3 antibody (top panels) or tag-specific antibodies (bottom panels, left: Flag, right: HA) in BT20 cells expressing either Flag-wtG3 or HA-neoG3, as indicated. DAPI was used to counterstain nuclei, GFP was expressed by the lentiviral vector used for the transduction. **c** EMSA assay performed with recombinant wtGATA3 or neoGATA3 and DNA fragment containing two GATAA motifs. **d** Reporter assay using the promoter regions of either *CDH1* or *CDH3* upstream of the luciferase cDNA. HEK293 cells were transiently transfected with the indicated constructs and luciferase activity was measured after 48 h. A GFP-expressing plasmid was co-transfected to normalize for transfection efficiency by western blotting (not shown). **e** Growth curve (left) and percentage of BrdU+ cells (right) measured in BT20 cells transduced with the indicated constructs. Data are represented as mean ± standard deviation of at least three independent experiments. Two-sided Student’s *t* test *P < 0.05, **P < 0.01.

Expression of HA-neoG3 or Flag-wtG3 in GATA3-negative BT20 (Fig. 1c) and MDA-MB-468 cells (Supplementary Fig. 6a), followed by cycloheximide treatment, revealed that neoGATA3 is markedly more stable than the WT protein (estimated half-life >16 h vs. 2 h, respectively) (Fig. 4a and Supplementary Fig. 6b). Importantly, this was not dependent on the tags, since we observed a similar effect with untagged proteins (Supplementary Fig. 6b). GATA3 stability is regulated by the proteasome through progesterone-induced phosphorylation of the S308 residue, missing in neoGATA3 [26]. Accordingly, treatment with the proteasome inhibitor MG132 increased the half-life of wtGATA3 but not of neoGATA3 (Supplementary Fig. 6c). Consistent with the IHC findings in tumors (Fig. 1d) neoGATA3 properly localized to the nucleus even in the absence of endogenous GATA3 (Fig. 4b and Supplementary Fig. 6d, e). However, unlike wtGATA3, purified neoGATA3—which lacks the C-terminal Zn finger essential for DNA binding—showed only a weak binding to an oligonucleotide containing two palindromic GATAA motifs in an EMSA assay (Fig. 4c and Supplementary Fig. 6f). Accordingly, neoGATA3 was unable to modulate the promoter activity of *CDH1* and *CDH3*, two known GATA3 targets [27, 28], in HEK293 cells using luciferase promoter reporter assays (Fig. 4d). Stable wtGATA3 expression in BT20 cells inhibited proliferation (*P* = 0.054) and BrdU incorporation (*P* = 0.012) (Fig. 4e) while neoGATA3 expression did not (Fig. 4e). Similar results were obtained in MDA-MB-468 cells (Supplementary Fig. 6g).

### NeoGATA3 interferes with the response to estradiol in ER + breast cancer cells

**Fig. 5:**
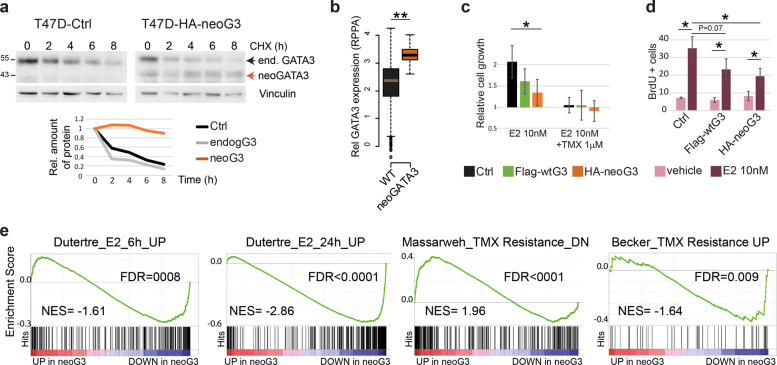
NeoGATA3 interferes with response to estradiol in ER + cells. **a** Western blot showing the expression of endogenous GATA3 or ectopically expressed neoGATA3 in T47D cells treated with 50 µg/ml CHX for the indicated time. Vinculin was used as loading control. Quantification of relative band intensity is shown at the bottom. The endogenous GATA3 band was quantified as well in the neoGATA3-transduced cells. The images are representative of at least three independent experiments. **b** RPPA data from the TCGA cohort showing GATA3 expression levels in tumors of the three groups of patients (WT *n* = 533, neoGATA3 *n* = 18). **c** Graphs showing the relative cell growth of T47D cells transduced with the indicated constructs, after 48 h in hormone-depleted (HD) medium followed by 72 h of treatment with E2 (10 nM) or with E2 (10 nM) and TMX (1 μM). All values are normalized to vehicle-treated cells of each experimental group. Data are represented as mean ± standard deviation of at least three independent experiments. Two-sided Student’s *t* test *P < 0.05 compared with treated Ctrl cells, ^#^*P* < 0.05 compared to vehicle-treated cells of the same experimental group. **d** Graphs showing the percentage of BrdU-positive nuclei in T47D cells transduced with the indicated constructs, after 48 h in HD medium followed by 24 h treatment with E2 (10 nM) or with vehicle. Data are represented as mean ± standard deviation of at least three independent experiments. Two-sided Student’s *t* test showed no statistically significant differences. **e** Enrichment plots for genesets related with estradiol stimulation and tamoxifen resistance in vivo. GSEA was performed on genes differentially expressed in METABRIC neoGATA3 tumors.

Because neoGATA3 mutations are exclusively found in ER + tumors (Supplementary Fig. 3b), we assessed neoGATA3 function in T47D and ZR75-1, two ER + /GATA3 + BC cell lines (Supplementary Fig. 7a). NeoGATA3 was more stable also in this context and the stability of endogenous GATA3 was not affected by the mutant (Fig. 5a, estimated half-life: >8 h vs 2 h). This is consistent with the observation that, in the TCGA-BRCA series, total GATA3 protein levels were significantly higher in the neoGATA3-mutant vs. WT tumors (*P* = 1.72e−07) [3] (Fig. 5b). Overexpression of wtGATA3 or neoGATA3 had no significant effect on proliferation and wound healing capacity of T47D and ZR75-1 cells (Supplementary Fig. 7b, c).

The patient-based transcriptomics analyses suggested that neoGATA3 modulates the ER-dependent program. Treatment of hormone-depleted control T47D cells with 17β-estradiol (E2) induced proliferation at 24–72 h, which was blunted by 4OH-Tamoxifen (TMX). By contrast, neoGATA3-expressing cells showed a significantly lower response to E2 (*P* = 0.025) (Fig. 5c, d). WtGATA3-overexpressing cells showed an intermediate phenotype (Fig. 5c, d). Similar, although less prominent, findings were made using ZR75-1 cells (Supplementary Fig. 8a, b). This observation was at odds with the enrichment of the two “Estrogen response” Hallmarks genes sets among overexpressed genes in the neoGATA3 tumors from patients. However, the Hallmarks gene sets do not discriminate between up- or down-regulated genes, therefore we performed GSEA on the METABRIC gene expression dataset, using an ad-hoc subset of estradiol- and TMX-related signatures selected from the MSigDb, with separate up- and down-regulated genes. The two signatures “Dutertre_E2_6h_UP” and “Dutertre_E2_24h_UP”, corresponding to genes upregulated in MCF7 BC cells upon E2 stimulation for 6 h or 24 h, respectively, were significantly enriched among the genes downregulated in neoGATA3 tumors, consistent with our in vitro observations (Fig. 5e). These data, together with the concordance between lower E2-induced proliferation in vitro and better outcome in patients, support the use of cell lines in vitro to investigate neoGATA3 function. Of note, gene expression signatures related to resistance to TMX in BC cell lines were also regulated in the neoGATA3 tumors (Fig. 5e).

### Genomic binding of ER is altered in neoGATA3-expressing cells

To understand how neoGATA3 interferes with the ER-dependent program, we checked the modulation of the ER protein upon hormone starvation and subsequent stimulation with E2 or TMX, which induce a reduction and an increase of ER, respectively [17]. NeoGATA3 expression did not affect the total ER levels in any of the tested conditions in T47D cells (Supplementary Fig. 8c, d).

**Fig. 6:**
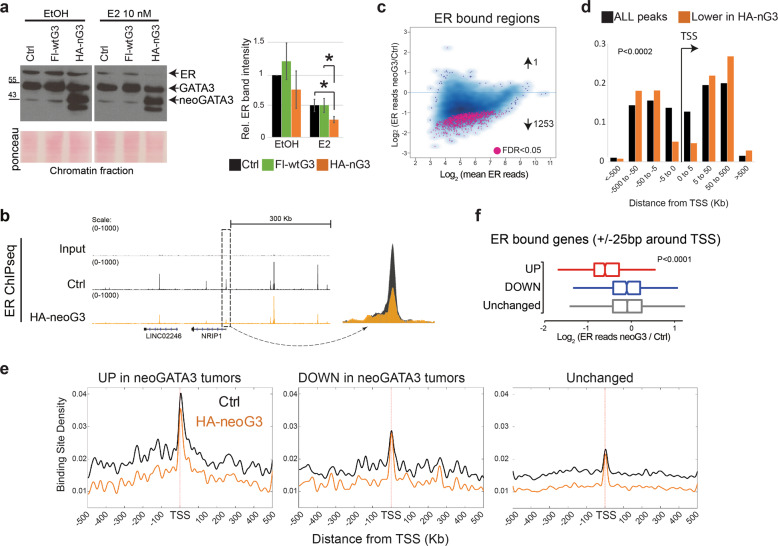
NeoGATA3 reduces the binding of ER to chromatin. **a** Western blot showing chromatin-bound ER and GATA3 in T47D cells transduced with the indicated constructs and treated as indicated for 24 h after 48 h in HD medium. Ponceau staining was used to assess equal loading. The quantification of the ER band intensity from three independent experiments is shown below. **b** Visualization of ChIP-Seq results in the genome browser. Magnification of the region in the box shows reduced ER signal in neoGATA3-expressing cells at the TSS of *NRIP1*. **c** Plot showing the differential binding of ER in Ctrl versus neoGATA3-expressing T47D cells. In pink are the peaks that show significantly reduced or increased ER enrichment in neoGATA3 cells calculated with DiffBind analysis. **d** Distribution of all peaks and peaks with reduced ER enrichment in neoGATA3 cells across the indicated intervals of distance from the TSS of the gene annotated to the peak. Chi-square test for differences in frequencies was applied. **e** ER binding density plot showing the enrichment of ER at ChIP-Seq peaks in Ctrl (black) and neoGATA3-expressing (orange) T47D cells considering genes that are upregulated, downregulated, or unchanged in the METABRIC ER+neoGATA3 tumors. **f** Box-plots show Log2 fold changes of normalized ER signals in neoGATA3-expressing T47D cells compared to controls. Signals were analyzed in regions ± 25 bp around TSS of genes that were upregulated (*n* = 999), downregulated (*n* = 921), or unchanged (*n* = 11537) in METABRIC neoGATA3 tumors. The signals are average values from ChIP-seq experiments in three biological replicates. Kruskal–Wallis *P* < 0.0001.

No difference was observed in chromatin-bound ER in hormone-starved T47D cells expressing neoGATA3 compared to Ctrl or wtGATA3-overexpressing cells. However, after stimulation with 10 nM E2 the drop in chromatin-bound ER was significantly more pronounced in neoGATA3-expressing cells compared to both Ctrl (*P* = 0.036) and wtGATA3-overexpressing cells (*P* = 0.016) suggesting that neoGATA3 interferes with the genomic binding of ER to some of its targets upon estrogen stimulation (Fig. 6a).

We then assessed the genomic localization of ER in Ctrl or neoGATA3-expressing T47D cells with ChIP-Seq, after 48 h of hormone starvation followed by stimulation with 10 nM E2. When merging replicates (Supplementary Fig. 9a) the total number of ER peaks was slightly reduced in neoGATA3 cells (7710) compared with Ctrl cells (9096) and 5591/7710 peaks (72.5%) were overlapping with peaks detected in Ctrl cells. Visual inspection of a set of peaks showed reduced signal in neoGATA3-expressing cells (Fig. 6b and Supplementary Fig. 9b–f). Differential binding analysis revealed that most of the common peaks had reduced ER enrichment in neoGATA3-expressing cells, with 1253 peaks showing significantly reduced binding, and only one peak with significantly higher binding (Fig. 6c). This is in accordance with an overall reduced binding of ER to the chromatin after stimulation with estrogen (Fig. 6a). Interestingly, 26.9% of all ER peaks but only 9.9% of those with reduced ER enrichment in neoGATA3-expressing cells were located within 5 kb from a TSS (Fig. 6d, *P* < 0.002) suggesting that neoGATA3 influences mostly the fine tuning of ER-dependent transcription, consistent with the subtle differences we observed in these cells.

To evaluate the extent to which our observations in T47D cells might reflect the scenario in patients, we analyzed the ER binding density in Ctrl and neoGATA3-expressing T47D cells at ChIP-Seq peaks located around the TSS of genes that were differentially expressed in neoGATA3 tumors from the METABRIC cohort. The binding density of ER peaks in neoGATA3-expressing cells was especially reduced on distal regions, independently from the resulting transcriptional effect (Fig. 6e). Interestingly, the binding density of peaks located at the TSS of genes that were either unchanged or downregulated in neoGATA3 tumors was maintained in neoGATA3-expressing T47D cells, but lower on the TSS of genes upregulated in neoGATA3 tumors (Fig. 6e, f). This suggests that neoGATA3 partially interferes with ER binding especially at enhancers and at the TSS of ER target genes that are repressed by ER. Of note, ER binding intensity in Ctrl T47D cells was generally higher on genes that were differentially expressed in neoGATA3 tumors compared to unchanged genes, supporting the use of this cellular model to understand neoGATA3 function in the context of ER-dependent transcription.

### NeoGATA3 interferes with progesterone-induced growth arrest

**Fig. 7:**
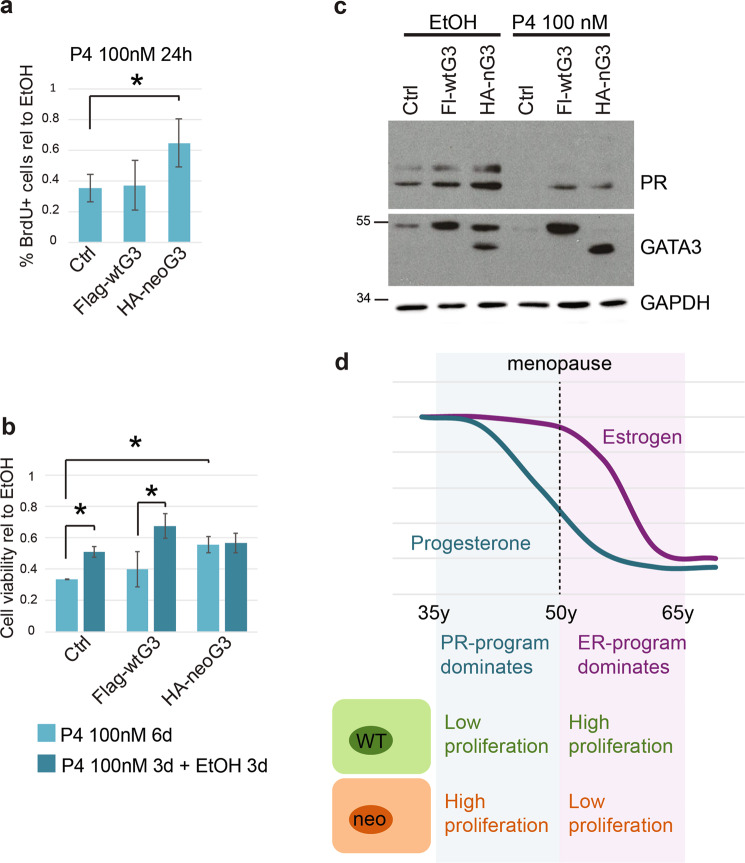
NeoGATA3 interferes with the PR-dependent growth arrest. **a** Graph showing the relative percentage of BrdU+ cells in the indicated cell population after 24 h treatment with vehicle or 100 nM P4. **b** Graph showing the relative cell viability measured with crystal violet staining of the indicated cell populations after vehicle-treatment, 6 days treatment with 100 nM P4, or 3 days with 100 nM P4 followed by additional 3 days in normal medium. Cells were kept in normal medium containing hormones. In **a**, **b** the results are normalized to the respective vehicle control and are shown as mean ± standard deviation of at least three independent experiments. Two-sided Student’s *t* test *P < 0.05. **c** Western blot showing expression of PR, wtGATA3, and neoGATA3 in T47D cells transduced with the indicated constructs and treated with progesterone (P4) (100 nM) for 24 h in normal medium. GAPDH was used as loading control. **d** The working model: neoGATA3 is a weak oncogenic driver with highly context-dependent functions. In a progesterone-rich environment, it interferes with the antiproliferative PR-driven program, whereas in an estrogen-rich context it blunts the pro-mitogenic ER-dependent response. Progesterone drops faster than estrogen at menopause, therefore it is possible that neoGATA3 mutations are enriched in premenopause patients because of this context-dependent opposite effects.

While the essential role of GATA3 for ER activity is well known, its relation with PR is much less studied. NeoGATA3 appeared to interfere with the transcriptional response to progesterone in tumors, especially in premenopause (Fig. 3e and Supplementary Fig. 5a). We therefore investigated the role of neoGATA3 in the PR-dependent program. As reported [6], hyperstimulation with P4 induced growth arrest in T47D cells, measured as BrdU incorporation after 24 h (Fig. 7a) and as cell viability after 6 days (Fig. 7b). This growth arrest was significantly reduced in neoGATA3-expressing cells both at 24 h (*P* = 0.041) and after 6 days (*P* = 0.007). When the P4-arrested cells were changed back to normal medium, with lower progesterone levels, for 3 additional days, both control and wtGATA3-overexpressing cells partially recovered proliferation, while neoGATA3-expressing cells remained arrested (Fig. 7b).

Interestingly, both GATA3 and neoGATA3 (in neoGATA3-expressing cells) were strikingly more abundant in the PR complex than in the ER complex, as shown by co-immunoprecipitation (Supplementary Fig. 10). Consistent with the lack of S308 in the neoGATA3, its expression was not reduced after treatment with 100 nM progesterone (P4) for 24 h, as opposed to endogenous GATA3 both in Ctrl and in neoGATA3-expressing cells (Fig. 7c). Interestingly, exogenous wtGATA3 was not reduced upon P4 treatment, possibly due to the overexpression of the protein from an ectopic promoter, evading the PR-dependent transcriptional inhibition [26].

## Discussion

*GATA3* is a paradigm of how genetic alterations in a given gene should not be lumped into a single class [5, 6]. Our work adds an important concept, namely that a single mutation might have distinct functions, depending on the context of the disease. This calls for an exhaustive functional characterization to refine our understanding of driver mutations roles and improve clinical application.

The *GATA3* X308_Splice mutation is predicted to produce a mutant protein lacking the second ZnFn and carrying a unique 44aa peptide (neoGATA3). Importantly, we identified five additional mutations predicted to generate a protein with partially or fully identical C-terminal peptides, supporting a selective pressure toward convergent oncogenic evolution. Here we provide formal proof, using a novel peptide-specific antibody, that neoGATA3 mutations are associated with less aggressive tumors and better outcome in patients. In vitro, neoGATA3 interferes with ER genomic binding upon estrogen stimulation, especially at enhancer regions. In addition, neoGATA3 interferes with the PR-dependent antiproliferative program in a progesterone-high context.

To understand the molecular function of neoGATA3 mutations in BC, we turned to an in vitro model. NeoGATA3 mutations are typically heterozygous, and the wtGATA3 protein is still expressed. Unfortunately, we did not manage to find or generate any cellular model carrying the mutation in the endogenous locus, therefore we decided to use lentiviral transduction of ER + cell lines. To control for increased abundance of total GATA3 proteins, we included wtGATA3-transduced cells. Indeed, the latter often showed an intermediate phenotype between Ctrl and neoGATA3-expressing cells, highlighting the limitations of our model. This might also suggest that part of the neoGATA3-dependent effects are simply due to its higher stability. Importantly, the major findings from our in vitro experiments were consistent with patient-based observations and we believe that our in vitro system is informative despite its limitations. Given the prevalence of neoGATA3 mutations in patients, the lack of models carrying them endogenously is striking and suggests that these alterations are likely weak drivers, conferring mild proliferative advantage only in specific contexts that are not easily reproduced ex vivo or in vitro.

The unique C-terminal peptide of neoGATA3 is a predicted neoantigen [21] proposed to be associated with increased T-cell-mediated immune response [29]. A neoantigen-elicited tumor clearance by the immune system would be an appealing explanation for the better outcome observed in patients carrying neoGATA3 mutations. However, we did not find evidence for this using multiple methods of analysis. The tumor immune milieu is indeed altered in neoGATA3 tumors, but the exact contribution of the immune infiltrates to their phenotype and whether/how neoGATA3 directly or indirectly influences the tumor microenvironment remains to be understood.

Our analyses of patient-derived datasets highlighted the ER-dependent transcriptome as highly altered in neoGATA3 tumors, in line with the known function of GATA3 as a crucial ER co-factor [11]. Indeed neoGATA3 expression reduced, but did not fully abrogate, the response to estrogen in two ER + BC cell lines. In particular, we observed a significant reduction of the chromatin-bound ER. In addition, ChIP-Seq revealed that the loss of ER binding was especially pronounced on regions far from the TSS, possibly enhancers. This suggests that neoGATA3 interferes mainly with the fine-tuning of ER-dependent transcription, consistent with the rather mild biological effects observed. As the C-terminal part of GATA3 is thought to modulate protein–protein interactions, the novel peptide contained in neoGATA3 could quench the formation of a functional DNA-binding ER complex or alter co-factors accessibility. Our analyses suggest that there might be a more obvious modulation of genes that are normally repressed by ER.

Having provided hints on the possible mechanism linking neoGATA3 to better prognosis in patients, we were puzzled by the fact that the neoGATA3 mutations, unfavorable for tumor cells, are highly selected during tumor evolution. We reasoned that there might be a context in which neoGATA3 grants a proliferative advantage. NeoGATA3 mutations are remarkably frequent among premenopausal patients, where estrogens and progestogens levels are relatively high and ER and PR have antagonistic effects through the modulation of shared targets [30]. GATA3 and PR are in the same protein complex [31] and PR reduces GATA3 both at transcriptional and post-translational levels, in particular by inducing phosphorylation of S308, which prompts GATA3 ubiquitination [26]. The strong selection of mutations abrogating this residue points toward a function in evading the PR-dependent antiproliferative program as we observed in T47D, where progesterone-induced growth arrest was less prominent in neoGATA3-expressing cells. Interestingly, wtGATA3 expression in T47D cells from an ectopic promoter evading PR-dependent transcriptional inhibition showed a tendency to reduce progesterone-induced growth arrest and 4 of the 5 neoGATA3-like mutations retain the S308 residue and are therefore likely degraded in response to progesterone. This would indicate that the increased stability of neoGATA3 is not the only explanation for the interference with PR function. Our data suggest that GATA3 is a crucial co-factor for both ER and PR and further—omics studies should be performed to assess its precise role in their transcriptional programs.

Intriguingly, the wtGATA3 was expressed at lower levels than neoGATA3 in T47D cells, yet wtGATA3-transduced cells often showed intermediate phenotypes between Ctrl-transduced and neoGATA3-expressing cells. This would suggest that an increase in GATA3 expression is sufficient to disrupt the balance between ER and PR programs, and that neoGATA3 is less efficient than the WT protein at doing this. Therefore, neoGATA3 behaves as a weak, inefficient, oncogenic driver.

In conclusion, our data suggest that neoGATA3 mutations are specifically selected in a molecular context where estrogen-driven mitogenic phenotypes are counterbalanced by progesterone-driven antiproliferative effects. In this scenario, the net output of neoGATA3 interference with both ER and PR programs seems to be a proliferative advantage. On the other hand, in a context where the ER-dependent program dominates, neoGATA3 confers a proliferative disadvantage and is associated with better patient outcome (Fig. 7d). The neoGATA3 mutations therefore represent a subtype of context-dependent weak driver mutations associated with distinct clinical features.

## Methods

### Patient samples and patient-related information

FFPE sections, DNA, RNA, and protein lysates from BC samples were obtained from the CRUK Cambridge, the Hospital INCLIVA (Valencia), and the Hospital Vall d’Hebron (Barcelona). Transciptomic data from TCGA and METABRIC were downloaded from cbioportal.org. All procedures were approved by the institutional Ethics Committees and informed consent was obtained from all patients.

## Supplementary information

Supplementary Material

Supplementary Figure 1

Supplementary Figure 2

Supplementary Figure 3

Supplementary Figure 4

Supplementary Figure 5

Supplementary Figure 6

Supplementary Figure 7

Supplementary Figure 8

Supplementary Figure 9

Supplementary Figure 10
