# Supplementary material for: The GATA3 X308_Splice breast cancer mutation is a hormone context-dependent oncogenic driver
Source: Oncogene. 2020 Jun 25;39(32):5455–67. doi: 10.1038/s41388-020-1376-3 (PMC7410826; doi:10.1038/s41388-020-1376-3)
Supplement: Supplementary file 1 — Supplementary Material [file 41388_2020_1376_MOESM1_ESM.docx]

**The GATA3 X308_Splice breast cancer mutation is a hormone context-dependent oncogenic driver**

Natascha Hruschka^1$^, Mark Kalisz^2$^, Maria Subijana^1^, Osvaldo Graña-Castro^3^, Francisco Del Cano-Ochoa^4^, Laia Paré Brunet^5,6^, Igor Chernukhin^7^, Ana Sagrera^2^, Aurelien De Reynies^8^, Bernhard Kloesch^1^, Suet-Feung Chin^9^, Octavio Burgués^10,11^, David Andreu^12^, Begoña Bermejo^10,13^, Juan Miguel Cejalvo^10,13^, Joe Sutton^7^, Carlos Caldas^9^, Santiago Ramón-Maiques^4^, Jason S Carroll^7^, Aleix Prat^5,6^, Francisco X Real^2,14^ and Paola Martinelli^1,2*#^

^1^Institute of Cancer Research, Medical University Vienna, Comprehensive Cancer Center, Vienna, Austria.

^2^Epithelial Carcinogenesis Group, Spanish National Cancer Research Centre-CNIO; CIBERONC, Madrid, Spain.

^3^Bioinformatics Unit, Spanish National Cancer Research Centre-CNIO, Madrid, Spain.

^4^Department of Genome Dynamics and Function, Centro de Biología Molecular Severo Ochoa (CSIC-UAM), Madrid, Spain

^5^Department of Medical Oncology, Hospital Clínic, Barcelona, Spain.

^6^Translational Genomics and Targeted Therapeutics in Solid Tumors, IDIBAPS, Barcelona, Spain.

^7^Cancer Research UK Cambridge Institute, University of Cambridge, Robinson Way, Cambridge, CB2 ORE, UK.

^8^Programme Cartes d'Identité des Tumeurs, Ligue Nationale Contre le Cancer, 75013 Paris, France.

^9^Department of Oncology, Cancer Research UK Cambridge Institute, University of Cambridge.

^10^INCLIVA Biomedical Research Institute, Valencia, Spain.

^11^Pathology Department, Hospital Clínico Universitario-CIBERONC, Valencia, Spain.

^12^Laboratory of Proteomics and Protein Chemistry, Universitat Pompeu Fabra, Barcelona, Spain.

^13^Oncology and Hematology Department, Hospital Clínico Universitario-CIBERONC, Valencia, Spain.

^14^Departament de Ciències Experimentals i de la Salut, Universitat Pompeu Fabra, Barcelona, Spain.

*Current address: Cancer Cell Signaling Department, Boehringer-Ingelheim RCV, Vienna, Austria.

#Corresponding author.

$These authors contributed equally

**Supplementary Methods**

**Patient-related information.** Data from the TCGA-BRCA cohort (clinical, gene expression, RPPA, and mutations) were obtained from the UCSC XENA browser (www.xena.ucsc.edu), while the data from the METABRIC cohort (clinical, gene expression, and mutations) were obtained from the cBio Cancer Genomics Portal ([www.cbioportal.org](http://www.cbioportal.org)). Survival analyses were performed with R; LogRank statistics was calculated with the Cox proportional hazard method. The MCP counter values were obtained from a previously published work [1]. The number of samples included in each analysis varies because not all samples had full clinical data and expression data.

**Patient samples.** Formalin-fixed paraffin-embedded sections of resected breast tumors, as well as DNA, RNA, and protein lysates from fresh tissue, together with the corresponding clinical data, were obtained from a cohort of 102 patients receiving surgery in the Hospital Clínico Universitario de Valencia/INCLIVA and one additional cohort of 100 patients from the Hospital Vall d’Hebron (Barcelona). FFPE from TMAs and full sections, as well as genomic DNA from one patient were obtained from the METABRIC cohort as described previously. All procedures were approved by the institutional Ethics Committees and informed consent was obtained from all patients.

The presence of the X308_Splice DNA mutation was assessed by PCR of the intron4-exon5 junction, followed by Sanger sequencing.

**Cell lines and treatments.** All cell lines used here are commercially available from ATCC. Cells were cultured in standard conditions (37 °C, 20% O_2_, 5% CO_2_) and periodically checked for mycoplasma contamination through PCR. HEK293FT cells were maintained in High Glucose DMEM supplemented with 10% FBS and 1% antibiotics (Pen/Strep). BT20, MDA-MB-468, T47D, and ZR75-1 were maintained in RPMI supplemented with 10% FBS and 1% antibiotics (all from Sigma-Aldrich). For the treatment with E2 and TMX in hormone-depleted medium, cells were kept for at least 48h in RPMI without phenol red, supplemented with 10% of charcoal-stripped FBS. T47D and ZR75-1 were authenticated by Eurofins Genomics. 17β-estradiol (E2), 4OH-Tamoxifen (TMX), progesterone (P4), cycloheximide (CHX), and the proteasome inhibitor MG132 were purchased from Sigma-Aldrich and dissolved in EtOH, which was then used as vehicle control. 5-Bromo-2’-deoxyuridine (BrdU) was purchased from Sigma-Aldrich, dissolved in water, and added to the cells for 2-4 hours at 50µM.

**Plasmids, transfection and lentiviral transduction**. The wild type GATA3 cDNA was a kind gift of C.M. Perou and J. Usary, the neoGATA3 cDNA was generated from it through site-directed mutagenesis. The CDH1 and CDH3 promoter reporter plasmids were a kind gift of A. Muñoz and J. Paredes, respectively. Promoter reporter plasmids were transfected into HEK293FT cells using jetPRIME DNA transfection reagent (Polyplus) following the instructions of the manufacturer. The pEGFP-C1 plasmid (Invitrogen) was co-transfected at 1:10 ratio for normalization. GATA3- and neoGATA3-expressing lentiviral plasmids were transfected into HEK293FT cells together with packaging plasmids with calcium-phosphate precipitation. Virus-containing supernatant from HEK293FT packaging cells was collected, filtered (0.45µm) and used to transduce epithelial cells.

**RNA-Seq re-analysis and identification of the neoGATA3 transcript.** Raw RNA-seq data were downloaded from CGHUB. RNA sequencing consisted of 48-50bp paired-end reads. To align reads to the human genome (GRCh37/hg19) TopHat-2.0.10 was used with Bowtie 1.0.0 and Samtools 0.1.19, allowing two mismatches and five multihits. Transcript assembly, estimation of abundance, and merging were performed with Cufflinks 2.2.1.

**Generation of a neoGATA3 gene expression signature.** To calculate the activity score, we downloaded the RNA-Seq raw data for the TCGA-BRCA cohort and identified genes differentially expressed between neoGATA3 tumors vs all other tumors using a SAM unpaired 2-class analysis. We selected up and downregulated genes with FDR<0.05. For each gene, we obtained a Dscore, which is a measure of the strength of its association. The signature was then used to classify samples in independent cohorts as follows: the Dscore of each selected gene was multiplied by its expression value and all the resulting values within each sample were summed to obtain a signature score. The highest the signature score, the more likely the sample has an expression profile consistent with the neoGATA3 mutant samples in TCGA.

**NeoGATA3-specific antibody production.** A peptide was synthesized based on the predicted neoGATA3-specific C-terminal sequence (PGEQGRPVRTVRPPQPHSGGGMPMGTLSAMPVGSTTSFTILTDP), including residues 309-352, plus a final Cys. The peptide was conjugated with KLH and used to immunize rabbits. Polyclonal antibodies were obtained following standard procedures.

**Immunoblotting.** Cells were lysed with Laemmli buffer and proteins were separated by SDS-PAGE and transferred onto nitrocellulose membranes. The following primary antibodies were used: anti-GATA3 (Cell Signaling #5852, or Cell Marque L50-823), anti-ER (Santa Cruz sc-543X or sc-8002), anti-Flag (Sigma-Aldrich F1804), anti-HA (BioLegend HA.11), anti-GAPDH (e-bioscience clone clone FF26A), and anti-vinculin (Sigma-Aldrich V9264). Anti-rabbit IgG or HRP-conjugated mouse IgG (Life Technologies) were used as a secondary antibodies. The chemiluminescent signals were detected with BioRad Chemidoc system or with Amersham films. Band intensity was quantified with ImageJ or with the BioRad Image Lab software.

**Immunofluorescence**. Cells were seeded on glass coverslips and fixed with 4% paraformaldehyde. After permeabilization with Triton 0.1%, coverslips were incubated with primary antibodies (GATA3, Flag, or HA), followed by Alexa-Fuor-conjugated fluorescent secondary antibodies (Life Technologies). Nuclei were counterstained with DAPI and coverslips were mounted with Agilent Fluorescent Mounting Medium. Images were acquired on a Zeiss LSM700 confocal microscope. For BrdU detection, the primary antibody (Sigma-Aldrich BU-33) was incubated together with 1µg/ml DNAse I (Sigma-Aldrich) in the presence of 6mM MgCl_2_.

**Immunohistochemistry**. Formalin-fixed paraffin-embedded sections were stained following standard procedures. Briefly, sections were de-paraffinized, re-hydrated, boiled in citrate buffer pH 6.0 for antigen retrieval, and incubated with 10% H_2_O_2_ in MetOH to quench the endogenous peroxidases. Afterwards, sections were incubated with primary antibodies recognizing GATA3 (Cell Signaling), neoGATA3 (home-made), and CD8α (DAKO C8/144B) overnight at 4 °C. HRP-conjugated secondary antibodies were from DAKO. 3,3-diaminobenzidine tetrahydrochloride plus (DAB+) was used as chromogen and nuclei were counterstained with hematoxylin.

**Cell proliferation, wound healing, viability assay**. To monitor cell growth, 4x10^4^ cells/well were seeded in 6-well plates in duplicates and counted every other day. For BrdU incorporation, cells were seeded on glass coverslips, treated as indicated, then fixed with paraformaldehyde and stained with anti-BrdU. For would healing experiments, cells were seeded in 12-well or 24-well plates and allowed to reach confluence. Then a scratch was done with a sterile 10µl pipette tip, the well was washed and the medium was replaced with serum-free RPMI. Microphotographs of at least 3 distinct regions of the wound were taken at the indicated time points and the open area was quantified with ImageJ. To assess cell viability upon E2 and TMX treatment, 5000 cells/well were seeded in 96-well plates in quadruplicates; the following day, the medium was changed to hormone-depleted RPMI and treatment with vehicle, E2, TMX, or the combination started after 48h. After 72h, cells were fixed with ice-cold methanol and stained with crystal violet. After extensive washing and drying, the dye was extracted with 1% SDS and the absorbance at 595 nm was measured on a Tecan microplate reader. For the treatments with P4 in estrogenic conditions, 50x10^4^ cells were seeded in 12-well plates in complete RPMI. Starting from the next day, the medium was changed daily, with the addition of either 100nM P4 or the corresponding amount of EtOH. After 72h, one set of wells was changed back to normal medium. Cells were fixed with ice-cold methanol after 6 days and stained with crystal violet. Cell viability was quantified as described above.

**Luciferase assay.** HEK293T cells were transfected with *CDH1* or *CDH3* promoter reporter plasmids, together with a GFP-expressing plasmid (pEGFP-C1). At the same time, empty pCDNA3 (Invitrogen), or pCDNA3 containing either wild-type GATA3 or neoGATA3 cDNA were introduced. Luciferase activity was measured with a luminometer, using a commercial luciferin solution (Promega) as a substrate. Values were normalized for transfection efficiency by checking GFP levels using western blotting.

**Cloning of wtGATA3 and neoGATA3 in pOPIN-B.** DNA sequences encoding the wild-type GATA3 region from residue 260 to 370 (wtGATA3) and the neoGATA3 region from residue 260 till the C-terminal end (neoGATA3) were PCR amplified from the full-length genes using the following oligonucleotides:

wtGATA3_Fw: aagttctgtttcagggcccgGGCAGGGAGTGTGTGAAC

wtGATA3_Rv: atggtctagaaagctttaTCAGCTAGACATTTTTCG

neoGATA3_Fw: aagttctgtttcagggcccgGGCAGGGAGTGTGTGAAC

neoGATA3_Rv: atggtctagaaagctttaTCAGGGGTCTGTTAATATTG

Amplicons were gel-purified and ligated into a pOPIN-B vector digested with HindIII and KpnI using In-Fusion technology (Clontech). The primer sequences in low case correspond to adaptors pairing with the linearized vector. This vector adds a poly-histidine tag and a cleavage site for the protease PreScission to the N-terminus of the protein. The correct insertions of the sequences in the plasmid were verified by sequencing.

**Protein production and purification.** *E coli* Rosetta (DE3) pLysS cells (Novagen) transformed with pOPIN-B plasmids encoding wtGATA3 and neoGATA3 were grown in autoinduction media supplemented with 50 µg/ml kanamycin and 34 µg/ml chloramphenicol for 8 h at 37 ºC followed by 21 h at 20 ºC. The cells, resuspended in buffer A (20 mM Tris-HCl pH 8, 0.5 M NaCl, 10 mM imidazole, 5% glycerol and 5 mM β-mercaptoethanol) with 0.4 mM Pefabloc (Merck), were disrupted by sonication. The clarified supernatant was applied onto a 5 ml HisTrap HP column (GE Healthcare) equilibrated in buffer A connected to an FPLC-Prime (GE Healthcare). Following extensive washing with buffer A supplemented with 34 mM imidazole, the protein was eluted with buffer A with 250 mM imidazole. The sample was dialyzed overnight in buffer B (10 mM HEPES pH 7.6, 0.1 M NaCl, 5 µM ZnSO4 and 1 mM DTT) and GST-tagged PreScission protease was added in the dialysis bag (1/20^th^ of the protein weight) to cleave the N-terminal tag. Then, the sample was loaded onto a 5 ml HiTrap-SP HP column (GE-Healthcare) equilibrated in buffer B, coupled to a 5 mL GST-Trap HP (GE-Healthcare) column to retain the protease. The untagged GATA protein eluted in a salt gradient at approximately 0.15 M NaCl and was concentrated in an Amicon 3K ultracentrifugation device (Millipore) and loaded onto a Superdex 75 10/300 column (GE-Healthcare) equilibrated in buffer B and connected to an AKTA FPLC system (GE-Healthcare). The protein eluted in a single peak and was concentrated up to 10 mg·ml^-1^ as mentioned above. The protein was supplemented with 20% glycerol, frozen in liquid nitrogen and store at -80ºC. All purification steps were carried out at 4 ºC and sample purity was assessed by SDS-PAGE.

**Electrophoretic Mobility Shift Assay (EMSA).** The following HPLC purified DNA oligonucleotides were purchased from Metabion:

DNA1_Fw: TGTCCATCTGATAAGAC

DNA1_Rv: GTCTTATCAGATGGACA

The oligonucleotides were resuspended in TE buffer (10 mM Tris-HCl pH 8.0, 1 mM EDTA) to a final concentration of 100 µM and were radiolabeled with T4 Polynucleotide kinase (PNK, Invitrogen) in a reaction containing 1 mM ATP-γ-^32^P (Perkin Elmer) for 2h at 37ºC. The reaction was diluted with TE buffer and loaded into an illustra MicroSpin G-25 Column (GE) to remove excess of free-radioactive ATP. Complementary strands were annealed by heating at 95ºC for 5 min, followed by slow cooling at room temperature. ^32^P-radiolabeled double stranded DNAs were stored at -20ºC. Samples for EMSA were prepared in a final volume of 20 µL, by mixing 100 nM of double stranded DNA1 or DNA2 with wtGATA3 or neoGATA3 at concentrations ranging from 0–0.4 µM, in a buffer containing 5 mM HEPES pH 7.6, 0.5 mM EDTA, 4 mM magnesium oxaloacetate, 50 mM potassium chloride, 10% glycerol, 1 mM DTT, 0.1 mg·ml^-1^ BSA and 2 µg·ml^-1^ salmon DNA. After 1 h incubation at room temperature, the samples were applied to a 6% acrylamide-TBE electrophoresis gel, ran in TBE 0.5x buffer at 150 V for 30 min at 4ºC. The gel was dried and exposed to a PhosphorScreen (GE-Healthcare) that was read in a Typhoon FLA 7000 (GE). Gel analysis was performed with ImageQuant software.

**Chromatin isolation.** Cells grown at 70-80% confluency were cross-linked in 1% formaldehyde at room temperature for 15 min, followed by 5 min quenching with glycine. Cells were harvested in PBS, centrifuged, and nuclei were isolated through the lysis in 0.34M sucrose/10% glycerol/10% Triton buffer followed by centrifugation. Nuclei were washed and further separated in nucleoplasm and chromatin fractions with 3mM EDTA/0.2mM EGTA buffer. The chromatin fraction recovered after centrifugation was sonicated and all fractions were resuspended in Laemmli buffer to be run on SDS-PAGE.

**ChIP-Seq.** Cells were grown until 70-80% confluence, treated 24h with 10nM E2, and cross-linked in 1% formaldehyde at room temperature for 10 min. After quenching, cells were harvested in PBS, nuclei were enriched with hypotonic solution, and lysed with 0.5% SDS. Chromatin was sonicated for 15 min in a Bioruptor water bath (Diagenode) using high intensity and 30”on/30”off cycles and then diluted to adjust SDS concentration to 0.1%. Immunoprecipitation was performed overnight at 4 °C with protein A/G agarose beads linked to anti-ERα (sc-543X) or rabbit IgG. Beads were washed 6x after incubation and the complex was eluted and de-crosslinked by overnight incubation at 65 °C. Eluted DNA was treated with proteinase K and purified with phenol-chloroform followed by isopropanol precipitation. All experiments were done as biological triplicates.

For sequencing, samples were diluted and pooled into NGS libraries. The E7645S NEBNext Ultra II DNA Library Prep Kit for Illumina and the E7335S NEBNext Multiplex Oligos for Illumina (Index Primer Set 1) were used according to the manufacturer's recommendations. Library concentrations were quantified with the Qubit Fluorometric Quantitation system (Life Technologies) and the size distribution was assessed using the Bioanalyzer (Agilent). Libraries were sequenced on Illumina HiSeq 3000/4000 instruments in 50-base-pair-single-end mode and base calls provided by the Illumina Real-Time Analysis (RTA) software were subse-quently converted into BAM format (Illumina2bam) before de-multiplexing (BamIndexDecoder) into individual, sample-specific BAM files via Illumina2bam tools (1.17.3 <https://github.com/wtsi-npg/illumina2bam>). Accession code: E-MTAB-9121.

**Peak calling and differential binding analysis.** Reads were aligned to the human genome (H. sapiens, UCSC hg19) using Bowtie2 [2] followed by exclusion of mapping quality scores < 30 by Samtools (v1.2) [3] and removal of duplicate reads with Picard (v2.6.0) and blacklisted regions with Bedtools (v2.13.3) [4]. Peaks were called with MACS2 (v.2.1.0) with settings: --extsize=300 and --q 0.05 [5]. Enriched regions were scored against matching input library. Differential binding of ER was analyzed with DiffBind (v2.6.6) [6] using the DEseq2 method without control input read counts. Differential peaks with FDR<0.05 were defined as significant. All TSS of differentially expressed and unchanged genes in METABRIC neoGATA3 tumors were extended by +/-25bp to generate TSS regions for 3 groups of genes: up-regulated, down-regulated and unchanged genes. Analysis of ER signals in those regions in T47D cells were based on the normalized values given in each bigwig file. The multiBigwigSummary function in Deeptools (v2.4.2) was used to compute the ER signal scores for each bigwig file in the 3 groups of regions. Regions with average signal scores of zero across all samples were omitted from the analysis. To assess differential ER binding, the Log_2_ fold change of the averaged normalized ER signal in neoGATA3 expressing cells versus control cells was plotted for each region. To assess overall differences in ER binding, we generated a single peak set for each condition with mergePeaks from HOMER software using the -d given flag [7] excluding peaks that did not overlap with any other peaks. Bigwig files were created with Deeptools, merged using bigWigMerge from UCSC tools (http://genome.ucsc.edu/) and displayed with IGV Browser.

**Binding site density (BSD) analysis.** To establish a functional relation between ER binding and differentially-expressed genes revealed in RNA-seq we evaluated the Binding site density relative to the active genomics features. BSD was calculated as sO/nG*corf where sO is the cumulative frequency, nG is number of tested features (genes) and corf is a density mean-correction factor. The cumulative frequency of chip locations was calculated as a sum of CHIP-seq sites overlapping 1kb bin size within +/-500kb window of genomic region with TSS of tested genes as a midpoint. The genes differentially expressed in the METABRIC neoGATA3 tumors were selected at FDR < 0.05.

**Gene Set Enrichment Analysis**. Differential gene expression was computed on the METABRIC and TCGA-BRCA datasets using the Comparative Marker Selection module of Genepattern, by comparing tumors carrying a neoGATA3 mutation and all other tumors. GSEA was then calculated on the ranked list of markers using the GSEA Preranked module and interrogating the Hallmarks and the C3-TFT Transcription Factor Targets gene set collections of the MSigDb of the Broad Institute. FDR <0.05 was considered significant.

**Statistical analyses.** The specific statistical test used for each analysis is indicated in the respective figure legend or table description. Statistical analyses were performed with R and R Studio. All statistical tests were confirmed as appropriate for the specific analysis and the distribution of the values. The distribution and variance of the experimental groups was estimated before deciding on the specific statistical test to be used.

1 Becht E, Giraldo NA, Lacroix L, Buttard B, Elarouci N, Petitprez F *et al*. Estimating the population abundance of tissue-infiltrating immune and stromal cell populations using gene expression. *Genome Biol* 2016; 17: 218.

2 Langmead B, Salzberg SL. Fast gapped-read alignment with Bowtie 2. *Nat Methods* 2012; 9: 357-359.

3 Li H, Handsaker B, Wysoker A, Fennell T, Ruan J, Homer N *et al*. The Sequence Alignment/Map format and SAMtools. *Bioinformatics* 2009; 25: 2078-2079.

4 Quinlan AR, Hall IM. BEDTools: a flexible suite of utilities for comparing genomic features. *Bioinformatics* 2010; 26: 841-842.

5 Zhang Y, Liu T, Meyer CA, Eeckhoute J, Johnson DS, Bernstein BE *et al*. Model-based analysis of ChIP-Seq (MACS). *Genome Biol* 2008; 9: R137.

6 Ross-Innes CS, Stark R, Teschendorff AE, Holmes KA, Ali HR, Dunning MJ *et al*. Differential oestrogen receptor binding is associated with clinical outcome in breast cancer. *Nature* 2012; 481: 389-393.

7 Heinz S, Benner C, Spann N, Bertolino E, Lin YC, Laslo P *et al*. Simple combinations of lineage-determining transcription factors prime cis-regulatory elements required for macrophage and B cell identities. *Mol Cell* 2010; 38: 576-589.

**Supplementary Figure legends**

**Supplementary Figure 1: The BC-specific X308_Splice** **hotspot *GATA3* splice mutation produces a truncated transcript and protein.** (**a**) qPCR analysis using primers amplifying both the wild type and the mutant GATA3 (left) or specific for the truncated neoGATA3 transcript (right) on tumor tissue. HEK293 cells transfected with either wtGATA3 or neoGATA3 were used as controls. (**b**) Western blot on total protein extract of tumor tissue, using an antibody that recognizes both wild type and mutant GATA3 (left) or with the neoGATA3-specific antibody (right). HEK293 cells transfected with either wtGATA3 or neoGATA3 were used as controls. (**c**) The 2-nucleotide insertion identified in the MB-0114 METABRIC tumor upon re-sequencing.

**Supplementary Figure 2: NeoGATA3 mutations are associated with markers of good prognosis.** (**a-d**) Distribution of tumor stage (**a**), grade (**b**), size (**c**), and PR status (**d**) among the three groups of patients in the METABRIC and TCGA-BRCA cohorts (METABRIC: stage WT n=648, neoGATA3 n=49, OtherMut n=104; grade WT n=891, neoGATA3 n=57, OtherMut n=133; size WT n=924, neoGATA3 n=59, OtherMut n=139; PR WT n=929, neoGATA3 n=59, OtherMut n=139; TCGA-BRCA: stage WT n=621, neoGATA3 n=20, OtherMut n=70; PR WT n=625, neoGATA3 n=21, OtherMut n=69). Fisher’s test Chi-square *P<0.05, **P<0.01.

**Supplementary Figure 3: NeoGATA3 mutations are prevalent in ER+ tumors and predict good outcome**. (a) Distribution of ER+ or ER- tumors among the indicated subgroups of the METABRIC and the TCGA-BRCA cohorts. AnyMut= any *GATA3* mutation. (**b**) Kaplan-Meier curves showing disease-free survival data of the TCGA ER+ cohort stratified according to the *GATA3* mutational status. (**c**) Graph showing the age at diagnosis of the METABRIC ER+ patients belonging to the three groups (WT n=1207, neoGATA3 n=66, OtherMut n=160). Fisher’s exact probability test was applied.

**Supplementary Figure 4: NeoGATA3 mutations are not associated with an immune cell infiltration.** (**a,b**) Gene expression levels of the indicated markers of T-lymphocytes, neutrophils, and M2 macrophages in tumors of the TCGA cohort, divided in the three groups according to the *GATA3* status (WT n=609, neoGATA3 n=20, OtherMut n=87). Mann-Whitney U-test *P<0.05, **P<0.01.

**Supplementary Figure 5: The neoGATA3 protein interferes with the PR-dependent program in tumors.** (**a**) Enrichment plots for two progesterone-related genesets among the differentially expressed genes in the METABRIC neoGATA3 patients compared to all other METABRIC ER+. (**b**) Gene expression data for the *PGR* gene in pre-menopausal METABRIC ER+ patients of the three groups (WT n=157, neoGATA3 n=21, OtherMut n=30). Mann-Whitney U-test *P<0.05, **P<0.01.

**Supplementary Figure 6: Expression of neoGATA3 in GATA3-negative BC cells.** (**a**) Western blot showing the detection of the Flag and HA tags in BT20 and MDA-MB-468 cells transduced with Flag-wtGATA3 (Flag-wtG3) or HA-neoGATA3 (HA-neoG3). Vinculin was used as loading control. (**b**) Western blot showing the protein level of Flag-wtG3 or HA-neoG3 expressed in the GATA3-negative MDA-MB-468 BC cells (top), or level of the untagged protein expressed in HEK293 cells (with undetectable endogenous GATA3) after treatment with 50 µg/ml CHX for the indicated time. Vinculin was used as loading control. (**c**) Western blot showing expression of Flag-wtG3 or HA-neoG3 in MDA-MB-468 after treatment with CHX, MG132, or both. Vinculin was used as loading control. (**d**) Representative images showing the negative controls of the experiment shown in Figure 3B. Ctrl-transduced BT20 cells are GATA3-negative, Flag-wtG3-transduced cells are HA-negative, HA-neoG3-transduced cells are Flag-negative. DAPI was used to counterstain nuclei, GFP was expressed by the lentiviral vector used for the transduction. (**e**) Immunofluorescence using the GATA3 antibody (top panels) or tag-specific antibodies (bottom panels, left: Flag, right: HA) in MDA-MB-468 cells expressing either Flag-wtG3 or HA-neoG3, or Ctrl-transduced cells, as indicated. DAPI was used to counterstain nuclei, GFP was expressed by the lentiviral vector used for the transduction. (**f**) Increasing amounts of neoGATA3 and wtGATA3 recombinant proteins used in the EMSA were loaded on a gel to check protein quality. (**g**) Growth curve of MDA-MB-468 cells transduced with the indicated constructs. Data are represented as mean ± standard deviation of at least three independent experiments.

**Supplementary Figure 7: Expression of wtGATA3 and neoGATA3 in luminal GATA3-positive BC cells.** (**a**) Western blots showing the expression of the indicated constructs in T47D and ZR75-1. **(b)** Growth curves of T47D and ZR75-1 cells transduced with the indicated constructs. Data are represented as mean ± standard deviation of at least three independent experiments. (**c**) Graphs showing the relative wound closure in a scratch assay performed with T47D and ZR75-1 cells transduced with the indicated constructs after 24h or 48h. Data are represented as mean ± standard deviation of at least three independent experiments.

**Supplementary Figure 8: NeoGATA3 interferes with the ER-dependent program *in vitro*.** (**a**) Graph showing the relative cell viability of ZR75-1 cells transduced with the indicated constructs and treated with E2 alone (10nM ) or in combination with TMX (1μM) for 72h after 48h in HD medium. (**b**) Graph showing the percentage of BrdU+ cells in ZR75-1 cells treated with E2 (10nM) for 24h. Data are shown as mean ± standard deviation of at least three independent experiments. Two-sided student’s T test *P<0.05, **P<0.01, #P<0.05 compared to the vehicle control. (**c**) Western blot showing the ER protein levels in T47D cells after 48h of hormone starvation followed by 24h of stimulation with E2 or TMX. Quantification of three independent experiments is shown. (**d**) Western blot showing ER expression in T47D cells transduced with the indicated constructs and treated 24h with vehicle (EtOH) or E2 (10nM) after 48h in HD medium. GAPDH was used as loading control. Quantification is shown.

**Supplementary Figure 9: ER ChIP-Seq in Ctrl and neoGATA3-expressing cells.** (**a**) Sample clustering using Principal Component Analysis showed consistency between biological replicates. (**b-f**) Examples of peaks showing equal or reduced ER binding in neoGATA3-expressing cells.

**Supplementary Figure 10: GATA3 and neoGATA3 are abundant in the PR complex and present in the ER complex.** Western blot showing the co-immunoprecipitation of GATA3 and neoGATA3 in the ER and PR complexes. Yellow asterisks indicate the two bands of PR (PRα and PRβ).

**Supplementary Tables**

**Supplementary Table 1:** *GATA3* mutations leading to a neoGATA3 protein (fully or partially concordant with the original X308_Splice mutant).

| **chromosomal location** | **gene location** | **change** | **C-ter** | **cBioportal nomenclature** |
| --- | --- | --- | --- | --- |
| chr10:8111432-8111434 | intr4-exon5 | TCA-->T | neoGATA3 (44 aa) | X308_Splice |
| chr10:8111513-8111513 | exon5 | T-->TGG | 20 aa | D335Gfs*21 |
| chr10:8106083-8106084 | exon4 | TA-->T | neoGATA3 | K302Sfs*53 |
| chr10:8111452-8111453 | exon5 | CA-->C | 40 aa | T315Rfs*40 |
| chr10:8111499-8111499 | exon5 | GA-->G | 25 aa | R329Gfs*26 |
| chr10:8111487-8111487 | exon5 | A-->AAA | 34 aa | not present (Q321Qfs*34) |

**Supplementary Table 2.** Cox model for disease-specific survival among METABRIC patients with ER+ tumors (n=1508).

|  | **Univariate analysis** | | | | **Multivariable analysis** | | | |
| --- | --- | --- | --- | --- | --- | --- | --- | --- |
|  |  |  |  |  |  |  |  |  |
| **Variables** | **HR** | **Lower 95%** | **Upper 95%** | ***P*-value** | **HR** | **Lower 95%** | **Upper 95%** | ***P*-value** |
| **Age (cont. variable)** | 1.02 | 1.01 | 1.03 | <0.001 | 1.01 | 1.00 | 1.02 | 0.011 |
| **Tumor size (cont. variable)** | 1.02 | 1.02 | 1.02 | <0.001 |  |  |  | <0.001 |
| **Stage** |  |  |  |  |  |  |  |  |
| T0 | 1.00 | - | - | - | 1.00 | - | - | - |
| T1 | 0.48 | 0.35 | 0.64 | <0.001 | 0.58 | 0.43 | 0.81 | 0.001 |
| T2 | 0.84 | 0.66 | 1.07 | 0.166 | 0.72 | 0.56 | 0.94 | 0.015 |
| T3 | 1.86 | 1.23 | 2.81 | 0.003 | 1.19 | 0.72 | 1.97 | 0.485 |
| T4 | 6.25 | 3.17 | 12.30 | <0.001 | 4.75 | 2.27 | 9.92 | <0.001 |
| **Grade** |  |  |  |  |  |  |  |  |
| I | 1.00 | - | - | - | 1.00 | - | - | - |
| II | 1.76 | 1.16 | 2.66 | <0.001 | 1.18 | 0.73 | 1.90 | 0.501 |
| II | 2.94 | 1.94 | 4.44 | <0.001 | 1.59 | 0.98 | 2.58 | 0.058 |
| **PR status** |  |  |  |  |  |  |  |  |
| Neg | 1.00 | - | - | - | 1.00 | - | - | - |
| Pos | 0.66 | 0.54 | 0.79 | <0.001 | 0.92 | 0.73 | 1.16 | 0.49 |
| ***GATA3* status** |  |  |  |  |  |  |  |  |
| WT | 1.00 | - | - | - | 1.00 | - | - | - |
| Other MUT | 0.64 | 0.45 | 0.91 | 0.014 | 0.65 | 0.43 | 0.98 | 0.040 |
| neoGATA3 | 0.26 | 0.13 | 0.52 | <0.001 | 0.46 | 0.23 | 0.94 | 0.034 |
| **PAM50 subtype** |  |  |  |  |  |  |  |  |
| Luminal A | 1.00 | - | - | - | 1.000 | - | - | - |
| Luminal B | 2.24 | 1.80 | 2.80 | <0.001 | 1.65 | 1.28 | 2.15 | <0.001 |
| Basal-like | 2.67 | 1.75 | 4.09 | <0.001 | 2.51 | 1.44 | 4.37 | 0.001 |
| HER2E | 2.39 | 1.73 | 3.30 | <0.001 | 1.86 | 1.20 | 2.87 | 0.005 |
| Normal-like | 1.34 | 0.95 | 1.90 | 0.096 | 1.50 | 1.00 | 2.26 | 0.048 |
|  |  |  |  |  |  |  |  |  |

**Supplementary Table 3.** Cox model for overall survival among METABRIC patients with ER+ tumors (n=1508).

|  | **Univariate analysis** | | | | **Multivariable analysis** | | | |
| --- | --- | --- | --- | --- | --- | --- | --- | --- |
|  |  |  |  |  |  |  |  |  |
| **Variables** | **HR** | **Lower 95%** | **Upper 95%** | ***P*-value** | **HR** | **Lower 95%** | **Upper 95%** | ***P*-value** |
| **Age (cont. variable)** | 1.05 | 1.04 | 1.06 | <0.001 | 1.05 | 1.04 | 1.05 | <0.001 |
| **Tumor size (cont. variable)** | 1.02 | 1.01 | 1.02 | <0.001 | 1.01 | 1.00 | 1.02 | <0.001 |
| **Stage** |  |  |  |  |  |  |  |  |
| T0 | 1.00 | - | - | - | 1.00 | - | - | - |
| T1 | 0.44 | 0.36 | 0.55 | <0.001 | 0.61 | 0.48 | 0.77 | <0.001 |
| T2 | 0.81 | 0.68 | 0.96 | 0.017 | 0.78 | 0.64 | 0.94 | 0.009 |
| T3 | 1.42 | 1.01 | 2.00 | 0.046 | 1.09 | 0.72 | 1.65 | 0.686 |
| T4 | 3.95 | 2.03 | 7.70 | <0.001 | 2.54 | 1.24 | 5.19 | 0.010 |
| **Grade** |  |  |  |  |  |  |  |  |
| I | 1.00 | - | - | - | 1.00 | - | - | - |
| II | 1.27 | 0.98 | 1.63 | 0.011 | 1.01 | 0.74 | 1.37 | 0.96 |
| II | 1.70 | 1.32 | 2.19 | <0.001 | 1.17 | 0.85 | 1.62 | 0.32 |
| **PR status** |  |  |  |  |  |  |  |  |
| Neg | 1.00 | - | - | - | 1.00 | - | - | - |
| Pos | 0.76 | 0.66 | 0.87 | <0.001 | 0.88 | 0.73 | 1.04 | 0.139 |
| ***GATA3* status** |  |  |  |  |  |  |  |  |
| WT | 1.00 | - | - | - | 1.00 | - | - | - |
| Other MUT | 0.74 | 0.58 | 0.93 | <0.001 | 0.77 | 0.58 | 1.04 | 0.089 |
| neoGATA3 | 0.33 | 0.22 | 0.51 | <0.001 | 0.58 | 0.36 | 0.92 | 0.020 |
| **PAM50 subtype** |  |  |  |  |  |  |  |  |
| Luminal A | 1.00 | - | - | - | 1.00 | - | - | - |
| Luminal B | 1.57 | 1.35 | 1.83 | <0.001 | 1.15 | 0.95 | 1.39 | 0.147 |
| Basal-like | 1.48 | 1.04 | 2.11 | 0.03 | 1.37 | 0.84 | 2.23 | 0.203 |
| HER2E | 1.55 | 1.22 | 1.97 | <0.001 | 1.25 | 0.90 | 1.76 | 0.183 |
| Normal-like | 0.98 | 0.76 | 1.27 | 0.90 | 1.22 | 0.89 | 1.68 | 0.206 |
|  |  |  |  |  |  |  |  |  |
