# Supplementary figures and images for: The GATA3 X308_Splice breast cancer mutation is a hormone context-dependent oncogenic driver
Source: Oncogene. 2020 Jun 25;39(32):5455–67. doi: 10.1038/s41388-020-1376-3 (PMC7410826; doi:10.1038/s41388-020-1376-3)

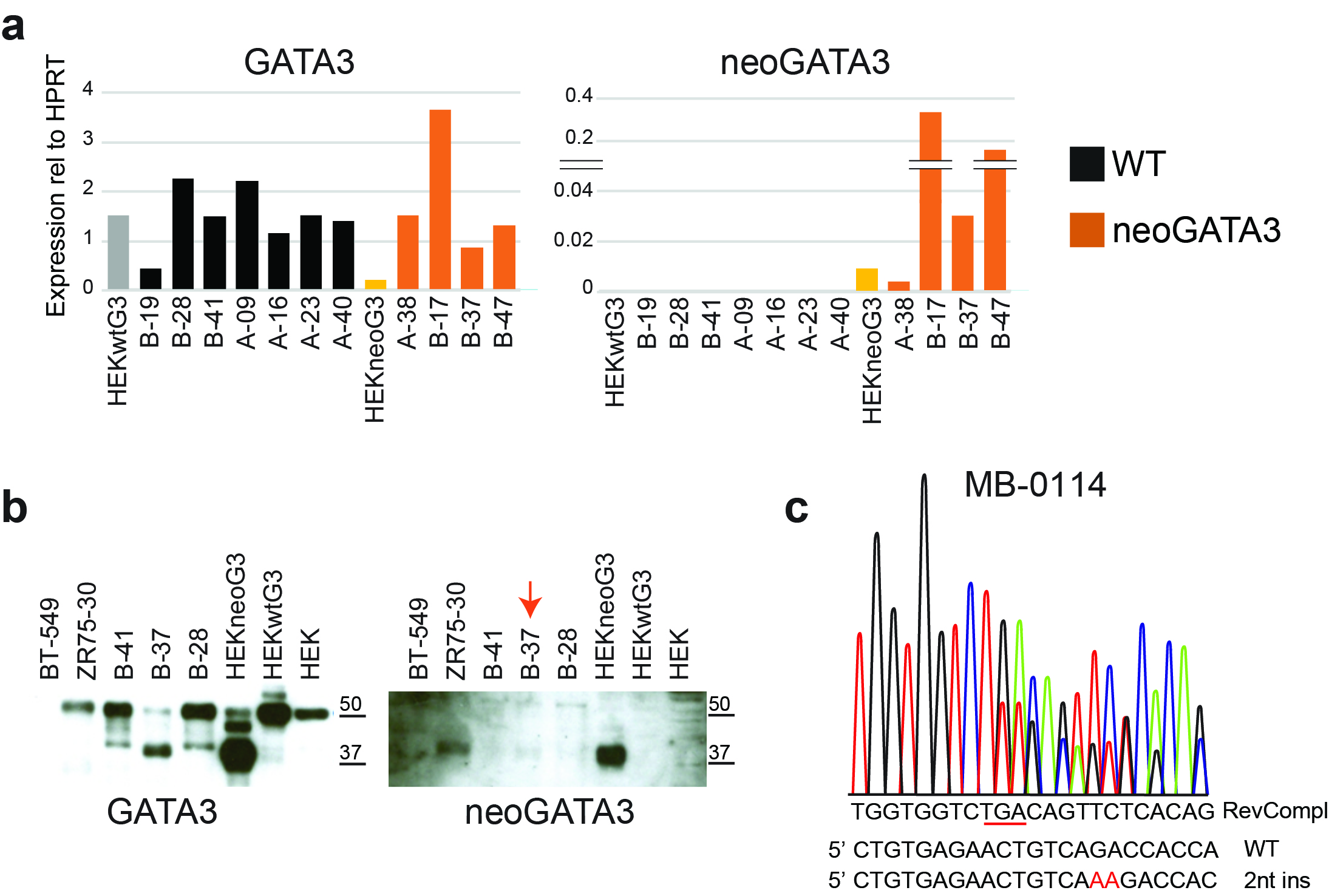

Supplement: Supplementary file 2 — Supplementary Figure 1 [file 41388_2020_1376_MOESM2_ESM.jpg]

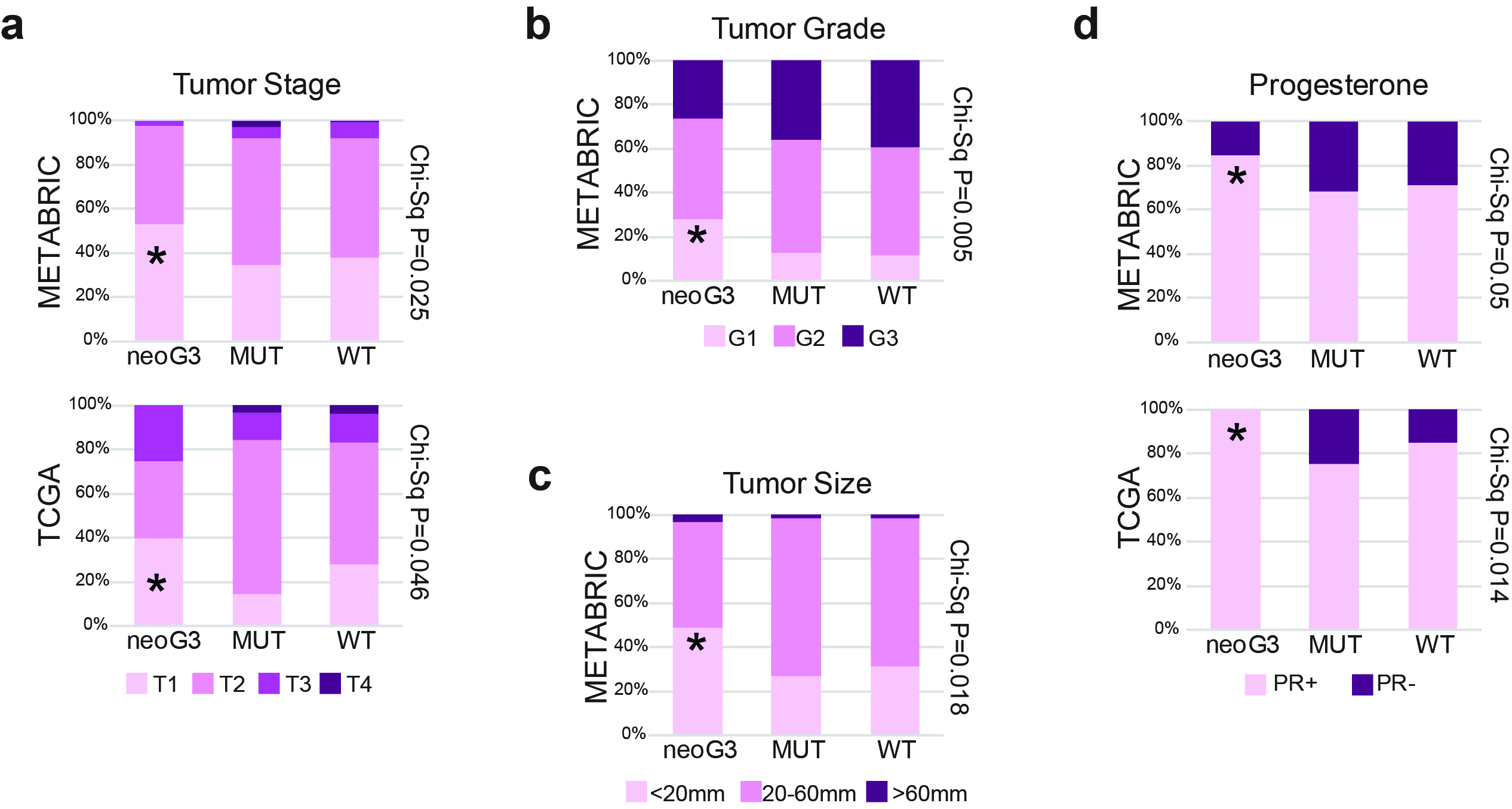

Supplement: Supplementary file 3 — Supplementary Figure 2 [file 41388_2020_1376_MOESM3_ESM.jpg]

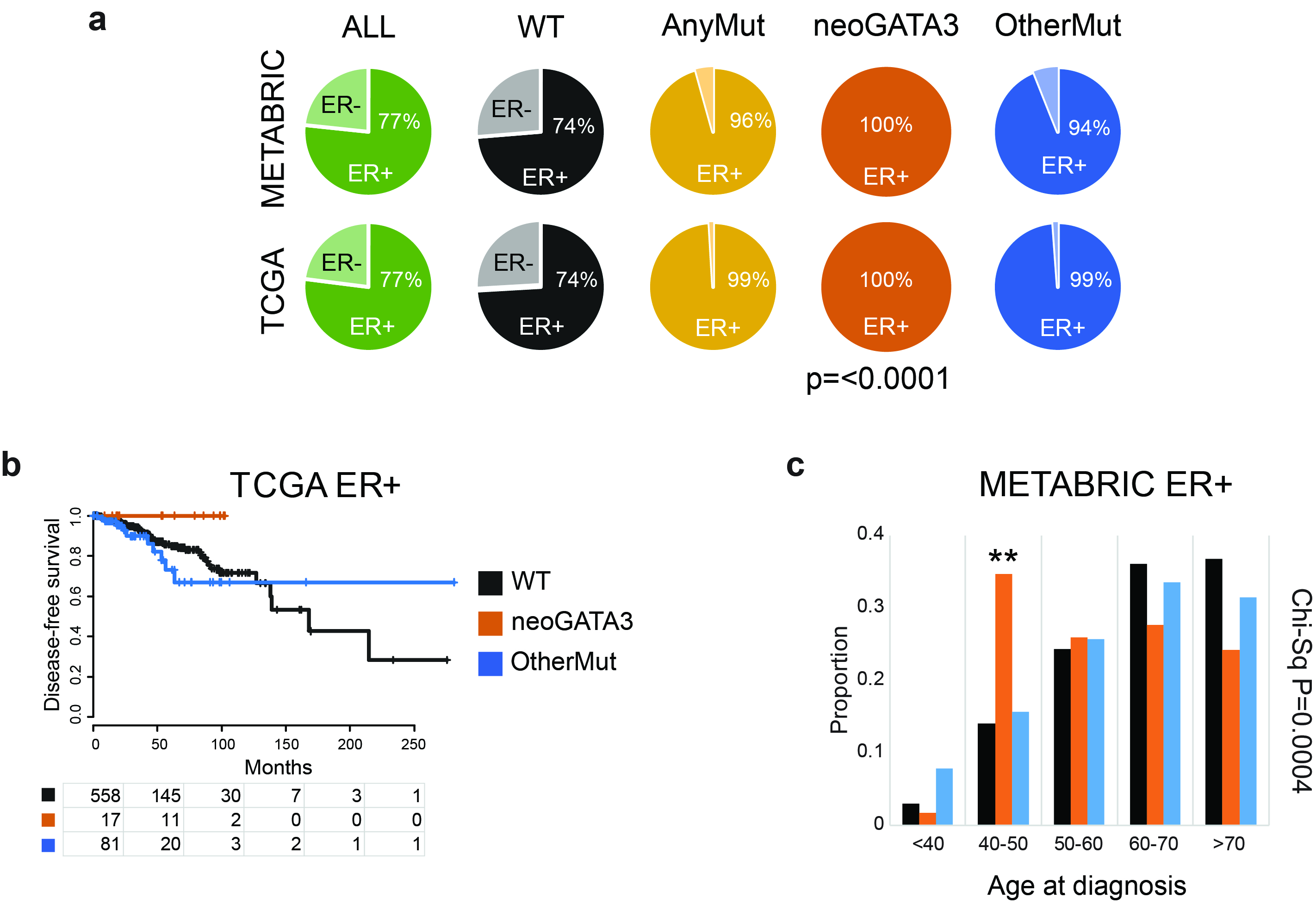

Supplement: Supplementary file 4 — Supplementary Figure 3 [file 41388_2020_1376_MOESM4_ESM.jpg]

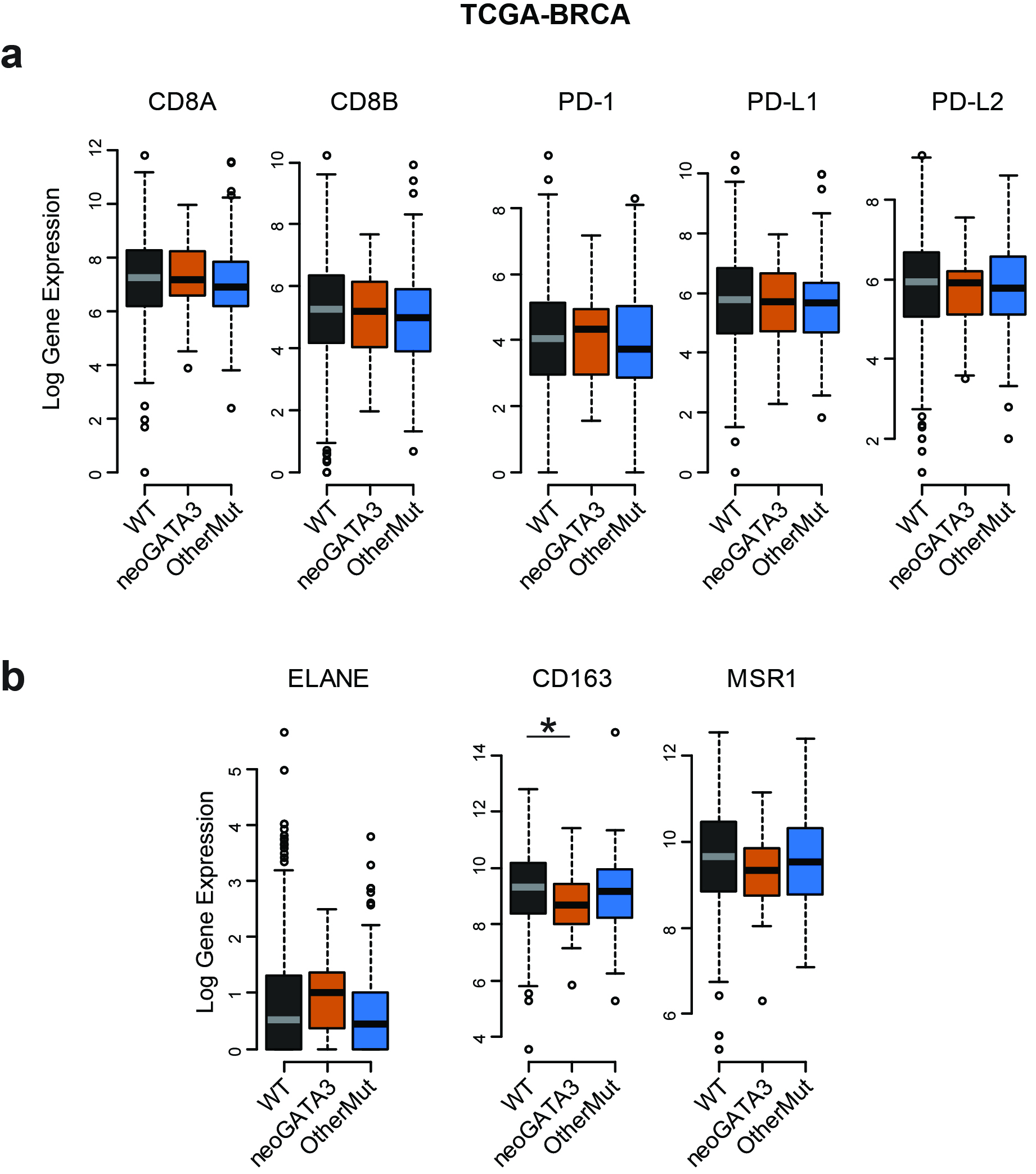

Supplement: Supplementary file 5 — Supplementary Figure 4 [file 41388_2020_1376_MOESM5_ESM.jpg]

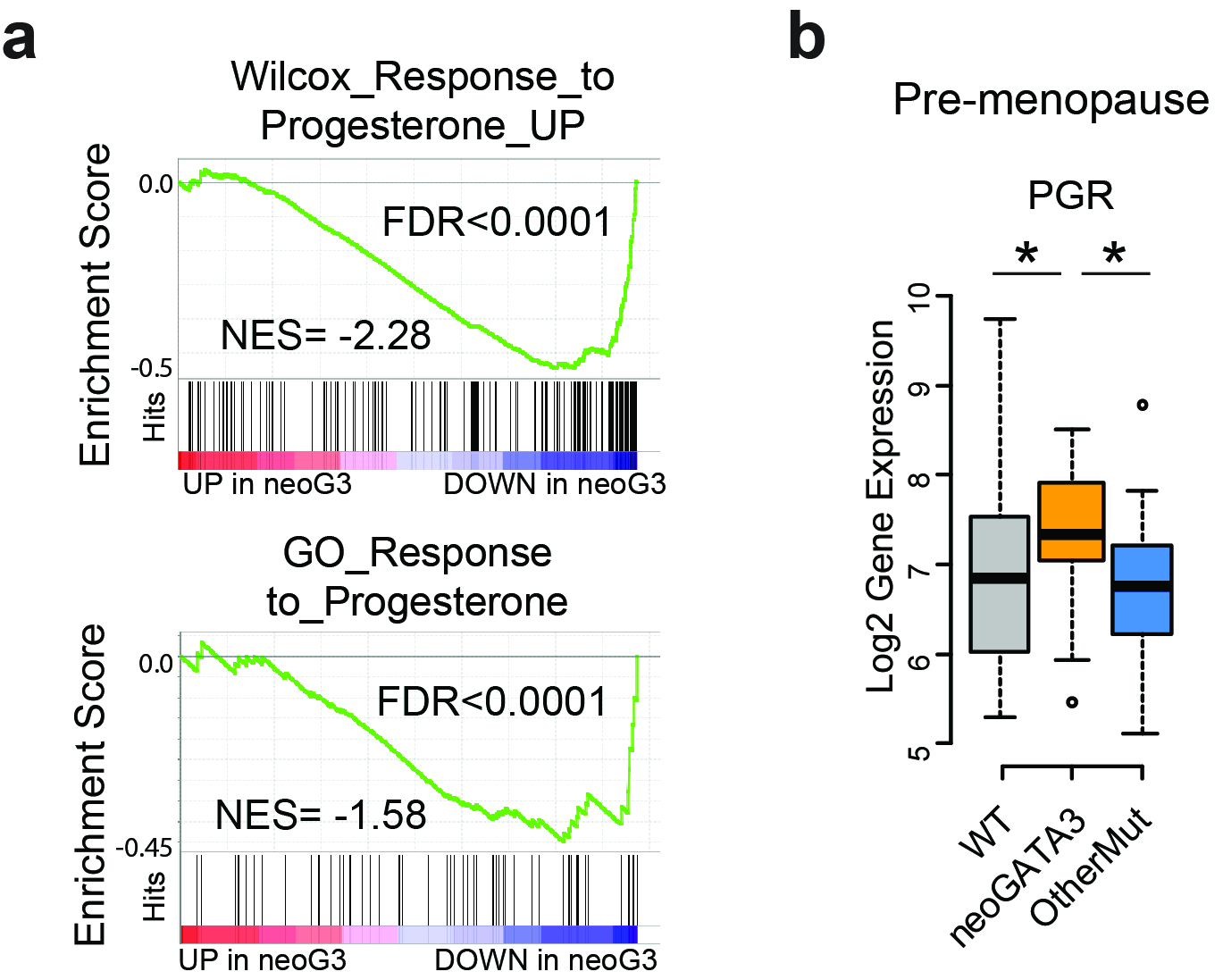

Supplement: Supplementary file 6 — Supplementary Figure 5 [file 41388_2020_1376_MOESM6_ESM.jpg]

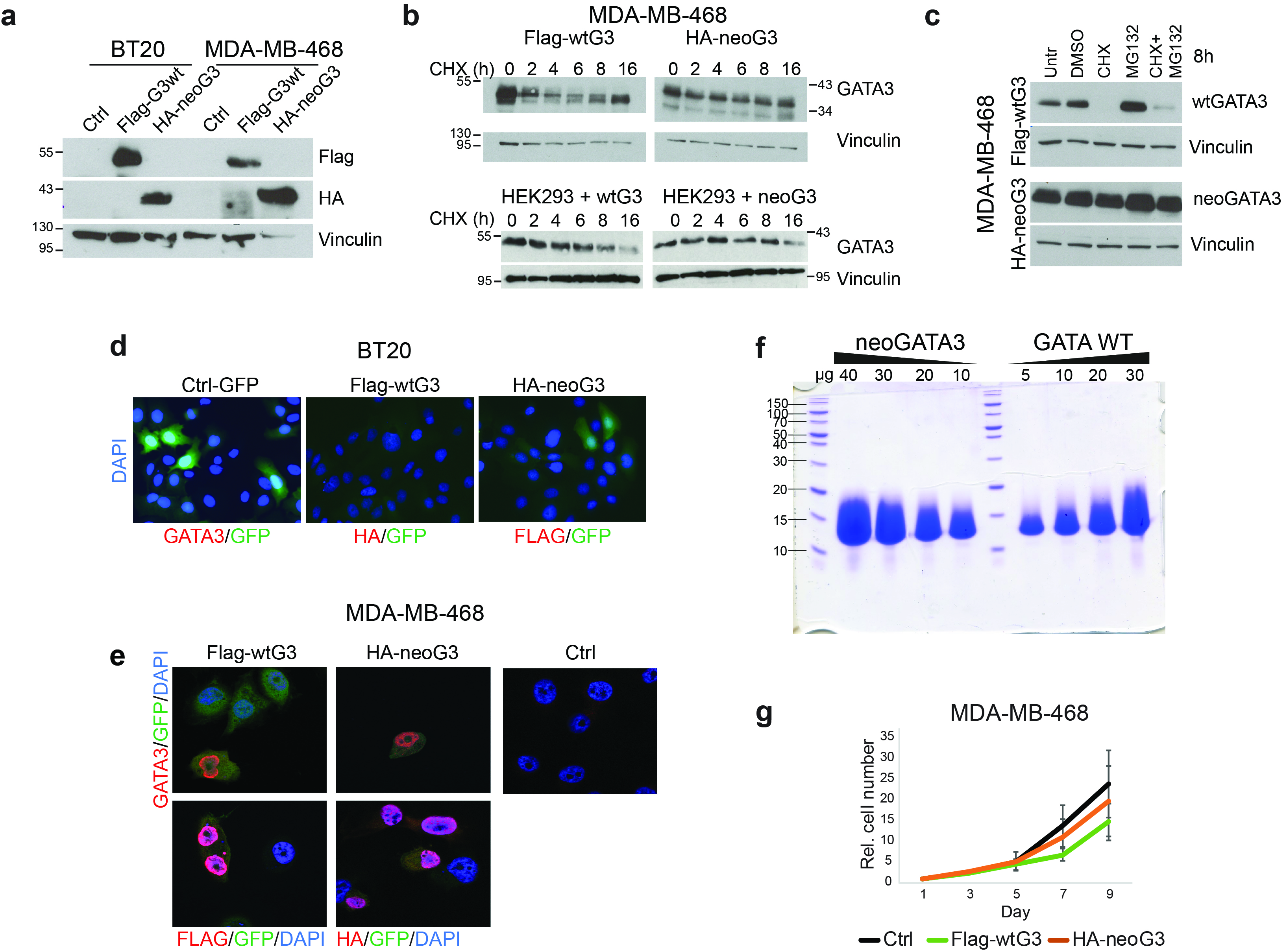

Supplement: Supplementary file 7 — Supplementary Figure 6 [file 41388_2020_1376_MOESM7_ESM.jpg]

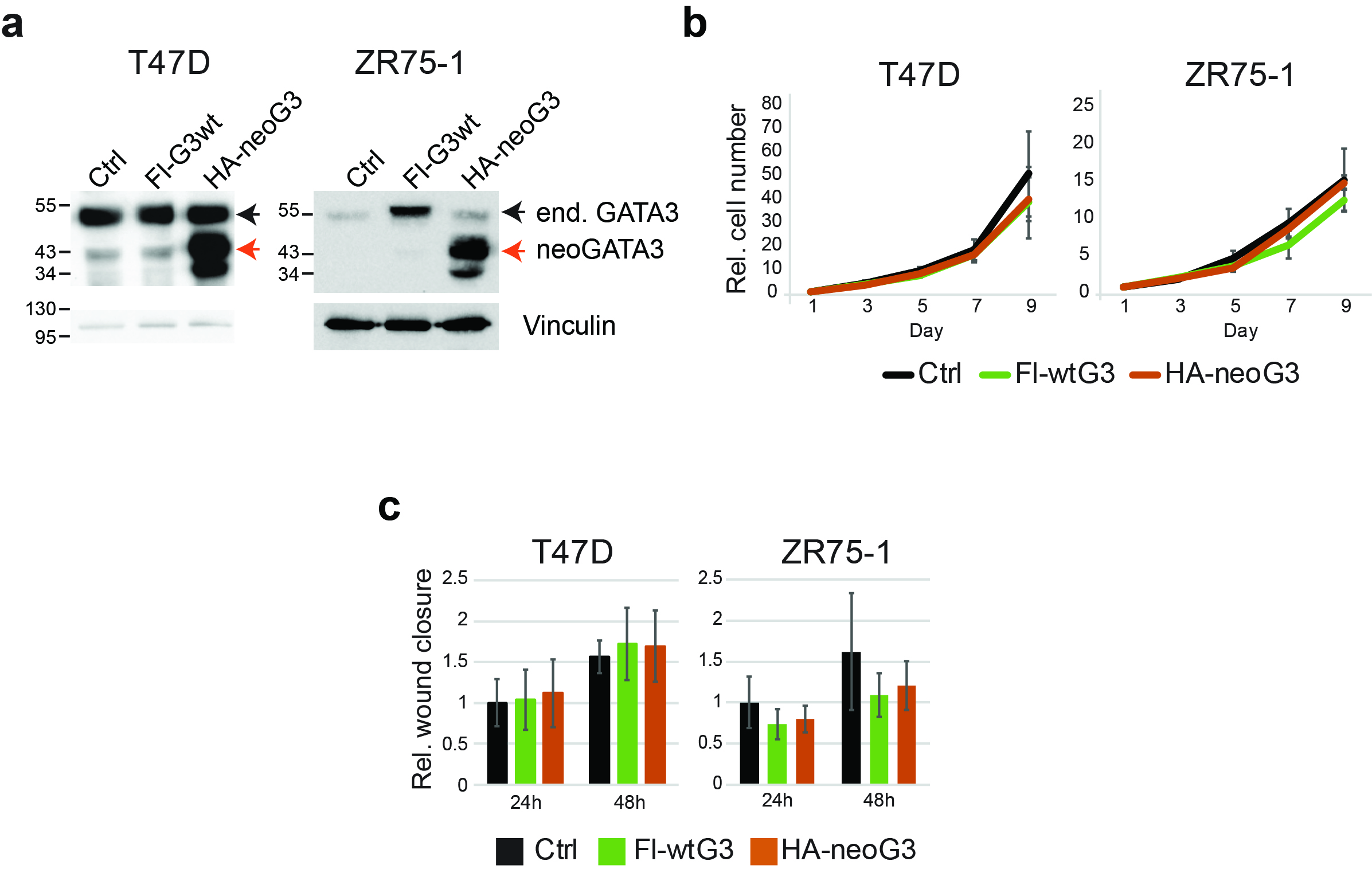

Supplement: Supplementary file 8 — Supplementary Figure 7 [file 41388_2020_1376_MOESM8_ESM.jpg]

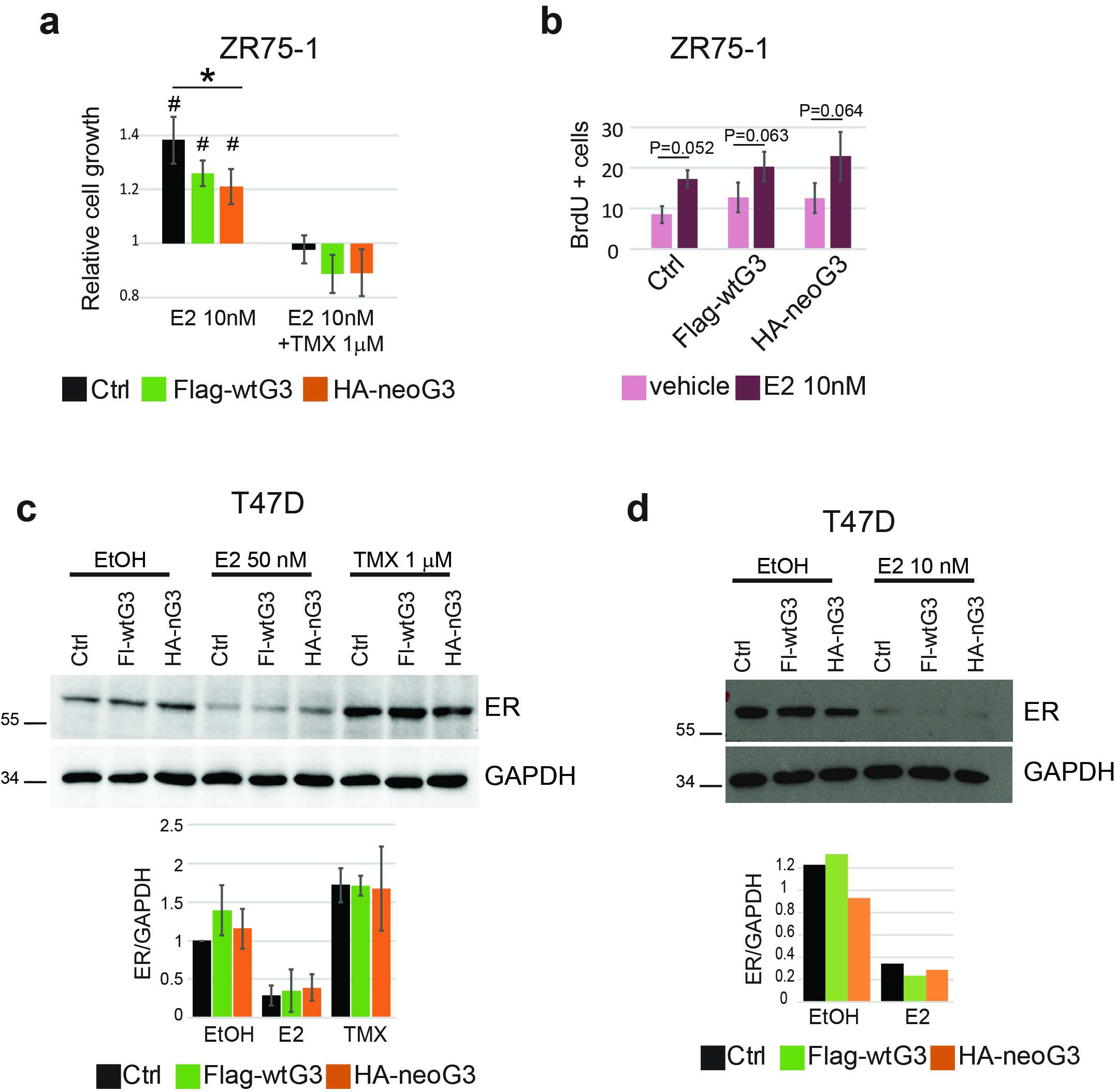

Supplement: Supplementary file 9 — Supplementary Figure 8 [file 41388_2020_1376_MOESM9_ESM.jpg]

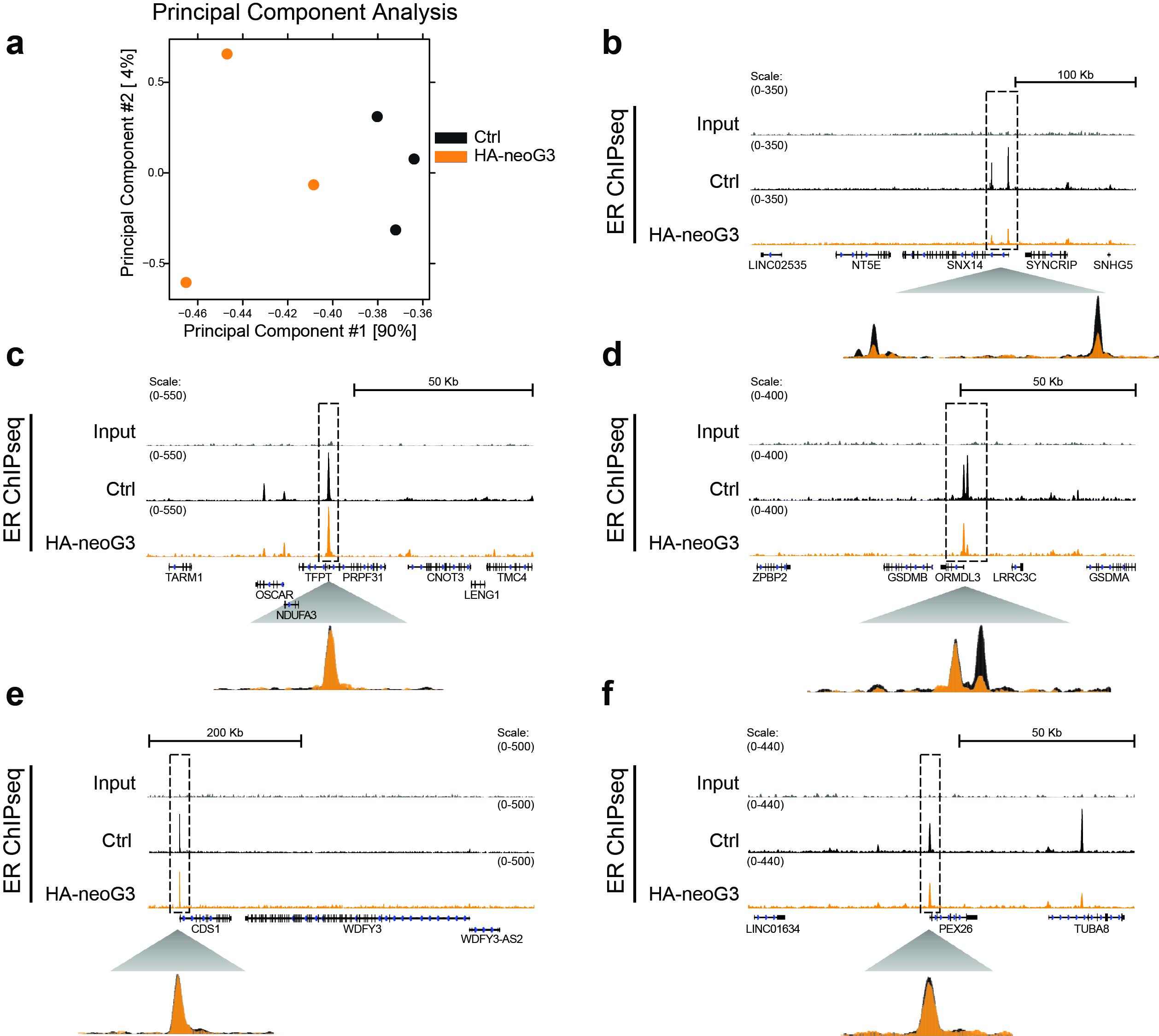

Supplement: Supplementary file 10 — Supplementary Figure 9 [file 41388_2020_1376_MOESM10_ESM.jpg]

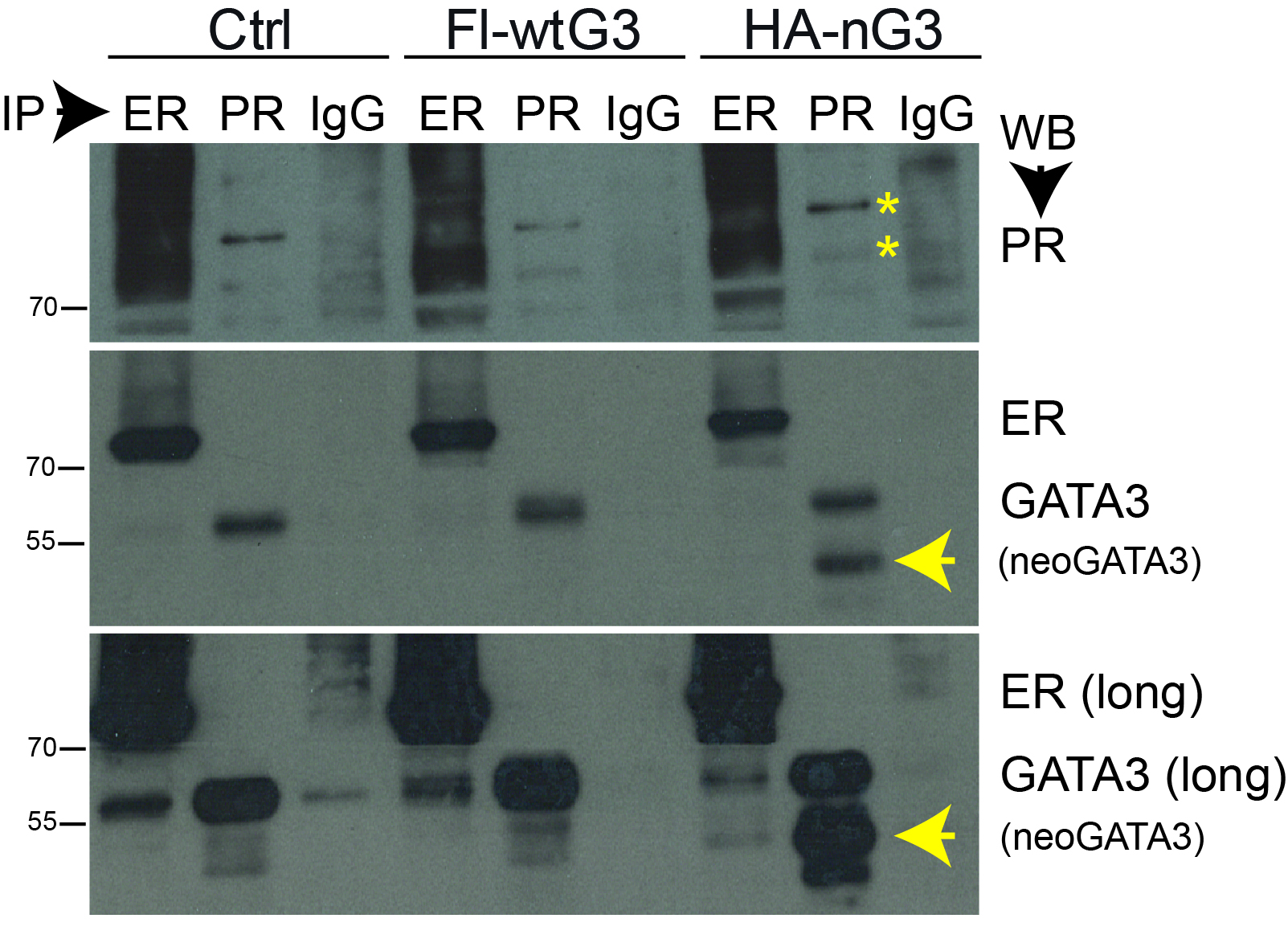

Supplement: Supplementary file 11 — Supplementary Figure 10 [file 41388_2020_1376_MOESM11_ESM.jpg]
